# Unlocking New Frontiers: Photo‐Isomerism and Magnetic Properties in Multifunctional Hetero‐Tetra‐Metallic Complexes

**DOI:** 10.1002/chem.202402601

**Published:** 2025-02-25

**Authors:** Ingrid Suzana, Jérémy Forté, Sébastien Pillet, El‐Eulmi Bendeif, Moritz Malischewski, Valérie Marvaud

**Affiliations:** ^1^ IPCM-CNRS, UMR 8232 Sorbonne Université 4 Place Jussieu 75252 Paris Cedex 05 France; ^2^ CRM^2^-CNRS Université de Lorraine 54506 Vandoeuvre-les-Nancy France; ^3^ Institut für Chemie und Biochemie – Anorganische Chemie Freie Universität Berlin Fabeckstraße 34-36 14195 Berlin Deutschland

**Keywords:** Hetero-poly-metallic, Multifunctionality, High-Spin molecules, Single-molecule magnet, Photo-isomerism

## Abstract

Hetero‐tetra‐metallic complexes, Fe_*NO*_CuLnCo (Ln=Gd, Tb, Dy), combining magnetic properties and photo‐isomerism, were obtained through the rational assembly of the photo‐switching nitroprusside anion Fe_*NO*_ with new magnetic Schiff base CuLnCo precursors. Herein, we describe the synthesis and characterisation of these compounds followed by a demonstration of their multifunctional character. Particularly noteworthy is the Fe_*NO*_CuTbCo complex which is one of the few examples of a photo‐isomerisable SMM.

## Graphical abstract

This study presents the synthesis, structural characterisation, and magnetic properties of hetero‐tetra‐metallic complexes, denoted as Fe_*NO*_CuLnCo, (with Ln=Gd, Tb, or Dy). These complexes mark a significant milestone as the first examples of polyfunctional compounds containing four different metallic ions, exhibiting dual properties: reversible photo‐isomerism and single‐molecule magnet behaviour.

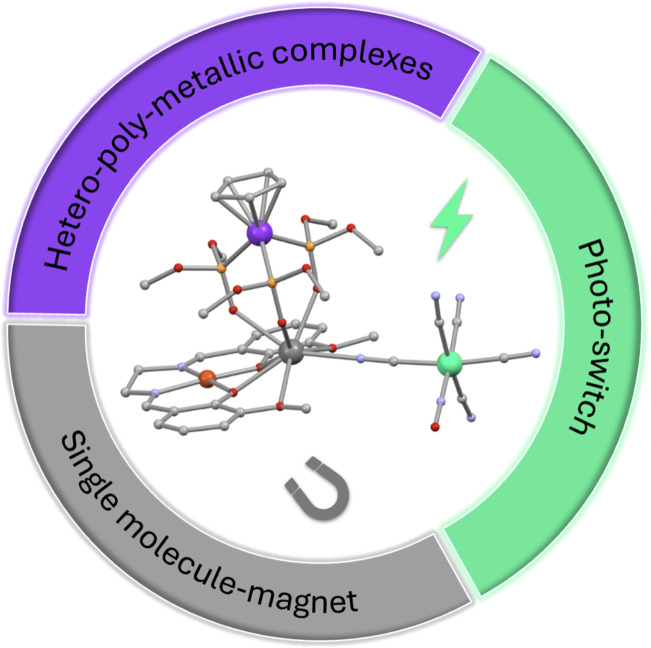

A new research focus in the field of molecular magnetism is the design of systems combining properties that may work in synergy, *i. e*. multifunctional materials.[^1^, ^2^, ^3^, ^4^] Synthetically, the incorporation of several functionalities into one molecule is rather challenging. However, the assembly of several functional subunits into a single molecular architecture represents a suitable approach.[^5^, ^6^] Such species combine the properties of each molecular subunit and may promote interactions between them resulting in synergistic effects. As subunits, inorganic species are good candidates since they provide multiple functionalities: magnetism, optics, redox etc. If chosen carefully, they are robust enough to keep their structure‐directing abilities when integrated into molecular edifices. And given the flexibility of coordination and organometallic chemistry, various chemical objects are within reach.

With the aim of designing multifunctional Single‐Molecule Magnets (SMMs), and especially photo‐switching SMMs, we have focused on hetero‐poly‐metallic species. They were first used to obtain High‐Spin Molecules (HSM) and SMMs,[^7^, ^8^, ^9^] but were then adapted to target multifunctional materials.[^10^, ^11^] Whilst increasing nuclearity improves the spin value,[^12^, ^13^, ^14^, ^15^] a great diversity of spin carriers within molecules of controlled shape may enhance the magnetic anisotropy essential to SMM.[^16^, ^17^, ^18^, ^19^, ^20^, ^21^, ^22^] When targeting multifunctional complexes, the idea then relies on the assembly of inorganic building blocks where each metallic ion brings its own function to the molecule. Hetero‐poly‐metallic species obtained through the building block approach thus combine both these aspects and may lead to multifunctional SMM.[^23^, ^24^] Although, the incorporation of more than two different metal ions within a single molecular assembly is not trivial. To date, only few hetero‐tri‐metallic assemblies have been reported,[^25^, ^26^, ^27^] and even less hetero‐tetra‐metallic species.^[28]^ Moreover, the use of building blocks often lead to extended networks and the obtaining of discrete entities requires great control over the molecular assembly and its topology.[^29^, ^30^, ^31^] Yet, we have overcome these two synthetic challenges using the “*complexes as ligands*” approach and yield unprecedented tuneable hetero‐tetra‐metallic species exhibiting dual properties.

The functional building blocks are the well‐known photo‐isomeric nitroprussides and the magnetic Schiff base derived dinuclear complexes. On the one hand, the valen Cu^II^Ln^III^ complexes, namely CuLn, where Ln is a lanthanoid, and valen the ligand (N,N’‐bis(3‐methoxy‐salicylidene)ethylenediamine), are excellent precursors for magnetic assemblies.^[32]^ This is mainly due to: (*i*) the lanthanoid high anisotropy owing to strong spin‐orbit coupling, (*ii*) the interactions between the 3*d* and 4*f* cations that limit the loss of magnetisation by Quantum Tunnelling Mechanism (QTM), (*iii*) the labile ligands which allow binding to other species. On the other hand, the nitroprusside ion, pentacyanidonitrosylferrate(II), Fe(CN)_5_NO^2−^ denoted hereafter Fe_*NO*_, is known for its light‐induced isomerism in the solid state.^[33]^ The nitrosyl group in the ground state, GS, may reach two metastable states MSI and MSII. The former MSI state corresponds to the isonitrosyl structure whilst the MSII state is associated to a side‐on conformation of the nitrosyl group.^[34]^ All these isomers correspond to energetic minima on the ground state potential surface, thus having a closed‐shell configuration.^[35]^ So far, most of the synthetic work has been directed on assembling the Fe_*NO*_ anion with metallic species to afford bi‐metallic networks. In such compounds, switching properties and modulation of the magnetic or optical properties were achieved through structural modifications.[^36^, ^37^]

To obtain discrete species featuring both the CuLn and Fe_*NO*_ subunits in a controlled manner, we have focused our effort on synthetic modifications of the CuLn precursors using the capping Kläui metallo‐ligand (η^5^‐Cp)tris(dimethylphosphito‐P)cobalt(III), [CpCo(PO(OMe)_2_)_3_]^−^ namely Co‐Kläui. On top of its oxophilic character and robustness, it has been reported by Lim *et al*. to magnetically isolate lanthanoid ions and induce a suitable coordination geometry for slow relaxation of magnetisation with various type of lanthanoid complexes (porphyrins, calixarenes, Schiff base ligands and more).[^38^, ^39^, ^40^, ^41^, ^42^, ^43^, ^44^, ^45^, ^46^, ^47^, ^48^] This strategy has yielded the CuLnCo (Ln=Gd^III^, Tb^III^, Dy^III^) trinuclear complexes where Co‐Kläui leaves only one available coordination site on the Ln^III^ ion. Although these complexes can be generated *in situ* for further reaction, they can be isolated as crystals suitable for SCXRD studies. Most importantly, these precursors retain their structure‐directing properties as no significant change around the CuLn pair is observed. All synthesis and characterisation of the hetero‐tri‐metallic precursors, including crystallographic data, are provided in the supporting information (SI).

Fe_*NO*_CuLnCo hetero‐tetra‐metallic complexes were therefore obtained in a one‐pot synthesis from the commercially available sodium Na_2_[Fe_*NO*_] salts and the Na[Co‐Kläui] salts as well as the dinuclear valen complexes [CuLn](NO_3_)_3_ (Ln=Gd^III^, Tb^III^, Dy^III^) whose syntheses are detailed in the SI. They were first obtained following a simple procedure, *method A*, illustrated in Scheme 1. The hetero‐tri‐metallic precursors CuLnCo^2+^ were prepared *in situ* in MeCN/H_2_O (3:1) by reacting one equivalent of Co‐Klaui^−^ with 1.25 equivalent of the dinuclear precursors CuLn^3+^. An aqueous solution of 1 equivalent of the Fe_*NO*_^2−^ ion was then added dropwise and stirred for a few minutes. The resulting solution was left to evaporate to yield neutral Fe_*NO*_CuLnCo complexes as red prism‐shaped crystals in 76–83 % yields. Remarkably, we found that tetraphenylarsonium(IV) chloride [As(Ph)_4_]Cl salts may slow down the crystallisation process and lead to crystals of better quality *(method B)*. First characterisations were performed by IR and ICP spectroscopies (SI). The IR spectra are noticeably identical and suggest the complex are isostructural whilst the ICP studies highlight the purity of the compounds.

**Scheme 1:**
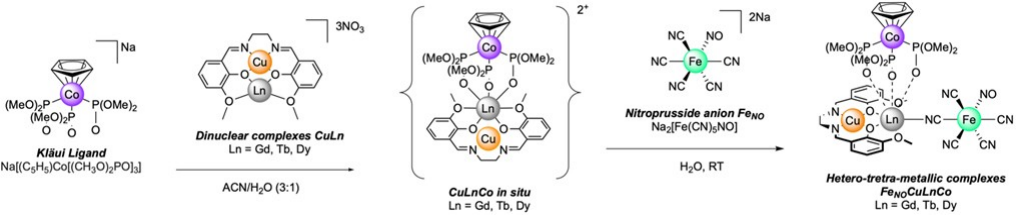
Synthetic procedure for obtaining the Fe_*NO*_CuLnCo complexes (with Ln=Gd, Tb, Dy).

SCXRD studies have been successfully performed on all three Fe_*NO*_CuLnCo complexes and the main crystallographic data are provided in the SI. They are isomorphous and isostructural: they crystallise in a triclinic system with the *P* $\bar{1}$ space group and two asymmetric units, containing the neutral Fe_*NO*_CuLnCo complex and 6 water molecules, are found in the unit cell. The cell parameters are roughly identical with average values of a=10.92 Å, b=14.75 Å, c=17.90 Å, and α=73.61 °, β=75.0 °, γ=68.81 °. The tetranuclear hetero‐tetra‐metallic complexes crystallise in the form of Schiff base compartmental ligand where the N_2_O_2_ and O_2_O_2_ cavities are occupied by a Cu^II^ ion and a Ln^III^ ion, respectively. The latter is capped by the O_3_ donor of Co‐Kläui and binds to the Fe_*NO*_ ion through a cyanide bridging ligand. The crystal structure of Fe_*NO*_CuGdCo (***1***) is represented in Figure 1. The geometry of the metal ions was determined using the program *SHAPE*.[^49^, ^50^]

**Figure 1:**
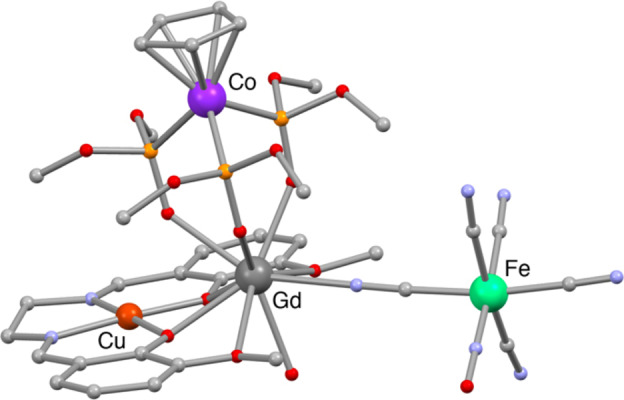
Crystallographic structure of the tetranuclear complex Fe_*NO*_CuGdCo (***1***). CCDC 2350763. Only metal ions are labelled while red, blue, yellow and grey spheres represent O, N, P and C atoms, respectively. Hydrogen atoms and water molecules are omitted for clarity.

The Cu^II^ and the Fe^II^ ions are found in a square planar and pseudo‐octahedral geometry, respectively. As described in the literature, Fe^II^ binds to the Gd^III^ ion through a cyanide bridging ligand in the cis‐position of the nitrosyl group.[^37^, ^51^] The lowest distortion parameter for the Gd^III^ ion was found for a muffin shape with a C_s_ symmetry. The GdO_2_Cu core is convex on Co‐Kläui side and the “butterfly” angle φ between the CuO_2_ and GdO_2_ planes is 31.16°. Considering Schiff base CuGd complexes, this value is rather large as they are known to range from 1.61° and 39.1°.[^52^, ^53^, ^54^] It is worth noting that the Cp group is not disordered in ***1***. This is a feature of the complexes obtained by *method B* where [As(Ph)_4_]Cl salts are added. For instance, this disorder is observable in the structure of Fe_*NO*_CuTbCo (***2***) which was first synthesised without the salts. Despite the neutral charge of Fe_*NO*_CuGdCo (***1***) and the absence of counter ions to spatially separate the molecules, the paramagnetic CuGd pairs are isolated in space. The shortest intermolecular metallic distance is observed between two Cu^II^ ions (6.69 Å). Similar crystallographic studies have been carried out on Fe_*NO*_CuTbCo (***2***) and Fe_*NO*_CuDyCo (***3***) complexes. The only difference concerns the butterfly angles φ which increase in value: φ (***1***, 31.16°) < φ (***2***, 31.73°) < φ (***3***, 31.90°). This trend has been observed in various isostructural Schiff base dinuclear complexes and is consistent with the lanthanoid contraction: r(Gd^III^) > r(Tb^III^) > r(Dy^III^).

Fe_*NO*_CuLnCo (Ln=Gd^III^, Tb^III^, Dy^III^) compounds have been studied by dc measurements to investigate the magnetic interactions involved between the spin carriers.^[55]^ Temperature dependence of the molar magnetic susceptibility *χ_m_* T products and the magnetisation curves of Gd^III^ (***1***), Tb^III^ (***2***) and Dy^III^ (***3***) analogues are presented in the SI. The *χ_m_* T product shows values of 8.321, 13.24 and 15.19 cm^3^ K mol^−1^ at room temperature. That is in good agreement with the theoretical values of 8.255, 12.195 and 14.545 cm^3^ K mol^−1^ for an isotropic Cu^II^ (S=^1^/_2_, *g*_Cu_=2.1) independent of the isotropic Gd^III^ (^8^S_7/2_, *g*_Gd_= 2) or anisotropic Tb^III^ (^7^F_6_, *g*_Tb_=^3^/_2_) and Dy^III^ (^6^H_15/2_, *g*_Dy_=^4^/_3_) ions.[^55^, ^56^] At 2.1 K, they reach maximum values of 10.8, 14.01 and 16.05 cm^3^ K mol^−1^. While the observed increase of *χ_m_* T reveals ferromagnetic interactions in ***1***, its fall and rise for ***2*** and ***3*** suggest this type of interaction but also significant anisotropy of the Tb^III^ and Dy^III^ ions and/or thermal depopulation of Stark sublevels.^[56]^

In Gd species ***1***, the nature of the interaction between the spin carriers was also extracted using the following Heisenberg‐Dirac‐Van Vleck Hamiltonian accounting for the Zeeman effect and the intra‐ as well as inter‐molecular interactions. The best fit gives the parameters: *J*_CuGd_ **=**2.30 cm^−1^, *g*=2.00 and *zJ’*=−0.02 cm^−1^. The coupling constant *J*_CuGd_ is in line with the literature considering the rather weak phenoxo‐mediated magnetic coupling between Cu^II^ and Ln^III^ ions,[^53^, ^54^, ^57^, ^58^] and the butterfly angle φ value. In addition, the *zJ’* term underlines negligible intermolecular interactions and thus shows the ability of Kläui capping ligand and nitroprusside anion to minimise these undesirable interactions. In contrast with ***1***, the thermal variations of *χ_m_* T of Tb^III^ (***2***) and Dy^III^ (***3***) species initially consist of a plateau between 300 and 150 K but then gradually decrease down to 25 K. On further cooling at 2.1 K, the *χ_m_* T values increase quite abruptly to reach values of 14.01 and 16.05 cm^3^ K mol^−1^. Here, the Cu^II^‐Ln^III^ interactions are expected to be ferromagnetic given the magneto‐structural correlations with the butterfly angles φ of 31.73 (***2***) and 31.90 ° (***3***).^[54]^

The dynamics of the magnetisation relaxation have been studied by ac magnetic measurements on polycrystalline Tb^III^ (***2***) and Dy^III^ (***3***) species to investigate their potential SMM behaviour. The frequency and temperature dependence of the ac susceptibility were measured under zero‐dc field and moderate fields (≤2 kOe) in the 2.5–10 K and 10–10k Hz ranges. At zero‐field, temperature and frequency‐dependent in‐phase *χ_m_’* and out‐of‐phase *χ_m_’’* components of ***2*** deviate significantly from zero (Figure 2). Specifically, frequency‐dependent out‐of‐phase components *χ_m_”* show maxima for each set of temperature and is in line with SMM behaviour at zero‐field. The relaxation times τ were extracted from the *χ_m_”* and *χ_m_’* components using a Debye model for each isotherm. An Arrhenius plot of these data shows the temperature dependence of the magnetic relaxation time *τ* and the linear regime suggests an Orbach mechanism. The energy barrier U_*eff*_ was therefore extracted using a thermally activated relaxation model: U_*eff*_=6.56 cm^−1^(9.4 K).

**Figure 2:**
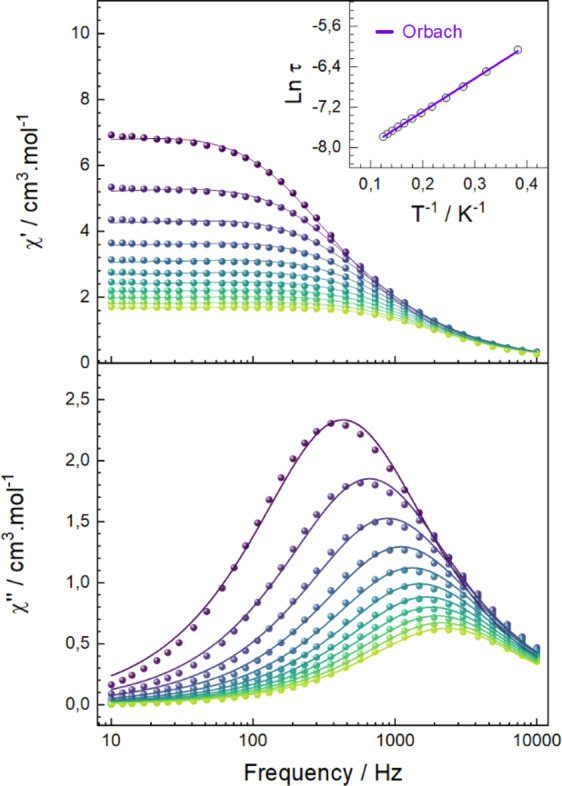
In‐phase (*χ*_m_’, top) and out‐of‐phase (*χ*_m_”, bottom) components of the ac magnetic susceptibility for Fe_*NO*_CuTbCo (***2***) under zero applied dc field at frequencies from 10 to 10k Hz and over the temperature range 2.5 (purple) to 7.5 K (light green). Data were collected in temperature increments of 0.5 K. The coloured lines represent fits to the data.

Regarding Dy^III^ species ***3***, ac measurements reveal the molecule is a field‐induced single molecule‐magnet (FI‐SMM). At zero dc‐field, out‐of‐phase components *χ_m_*” show that there is no barrier to spin reversal. Under an applied field, frequency‐dependent out‐of‐phase components *χ_m_*” exhibit maxima for each set of temperature until 5 K and indicates slow relaxation of magnetisation. As complexes ***2*** and ***3*** are isostructural, the difference in behaviour between the two oblate Dy^III^ and Tb^III^ ions is likely originating either from their respective Kramer or non‐Kramer character or, as reported in the literature, from weaker magnetic interactions with the Cu^II^ ions.[^54^, ^59^, ^60^] Here, the interactions are expected to get weaker from the Gd^III^ to the Dy^III^ ion given that the polarisation effect, essential to the magnetic interaction, increases with the number of unpaired electrons in the 4*f* orbital.[^54^, ^60^] In addition, magnetic interactions in dinuclear 3*d*‐4*f* complexes are correlated with the butterfly angle LnO_2_M (φ): the larger the angle, in the angle, the weaker the interactions. In Fe_*NO*_CuLnCo, these angles range from 31.16° (***1***), 31.73° (***2***) to 31.90° (***3***), and the magnetic interaction values most likely follow this trend.

To evaluate the switchable properties of Fe_*NO*_CuLnCo complexes, all three compounds have been studied by FT‐IR spectroscopy under *in situ* excitation. This method represents an excellent choice to investigate qualitatively the Fe_*NO*_ photo‐activity since the GS, MSI & MSII states exhibit characteristic stretching bands. The experimental procedure is as followed: *i)* collection of spectra upon cooling, *ii)* optical excitations at 10 K and, *iii)* collection of spectra upon heating. Our preliminary experiments consisted in first recording UV spectra and screen the MLCT absorption band. And second, performing FT‐IR experiments under irradiation with different light sources and exposure times. More details on the experimental procedure are provided in the SI. Photo‐excitation with 405 nm laser is the most efficient and was used for in‐depth studies on all three valen species. Here, we detail the investigations on the Dy^III^ species ***3*** while experimental data of Gd^III^ (***1***) and Tb^III^ (***2***) species are given in the SI.

Upon cooling, spectra were collected in temperature increments of 50 K and their superimposition underlines the lack of significant changes (SI). At 10 K, irradiation of the sample was performed with a 405 nm laser for 30 min. Spectra of the excited species were then collected after irradiation and thermalisation of the sample. The superimposition of the spectra in the nitrosyl region – before (ground state, GS) and after irradiation (excited state, ES) – is represented in Figure 3. In GS, the NO stretching vibration is measured at 1910 cm^−1^, corresponding to the nitrosyl configuration Fe−N=O. In ES, a weak but nevertheless significantly higher than the background additional band is detected at 1770 cm^−1^. This 140 cm^−1^ shift is the signature of an isonitrosyl configuration Fe−O=N^+^ (MSI) by comparison with experimentally characterised photo‐isomerism process in other nitrosyl Fe complexes.[^61^, ^62^] The weakness of the band indicates a low population of MSI state and is in accord with the reported values in lanthanoid‐containing nitroprusside materials.^[37]^ It is important to stress that the experimental conditions mainly provide a qualitative study of the nature of the metastable state. In addition of possible low 405 nm light penetration, the samples have been ground and pressed into pellets while the highest conversions are achieved on crystals.^[63]^

**Figure 3:**
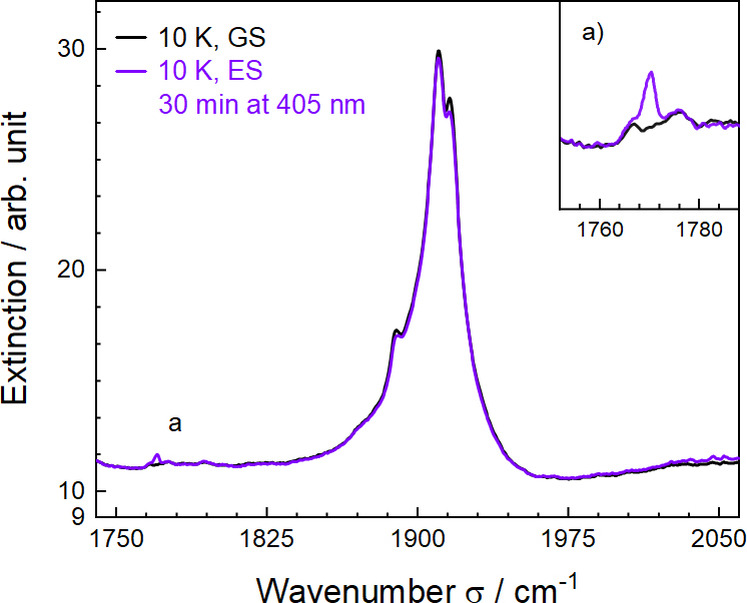
InfraRed spectra superimposition of Fe_*NO*_CuDyCo before excitation and after 30 min of irradiation at 405 nm.

In order to probe optical reversibility, the excited sample was further irradiated with a 1064 nm laser for 10 min. The superimposition of the two spectra – excited and after irradiation (1064 nm) at 10 K – is given in the SI. This shows unambiguously the optical reversibility of the system as the band of the isonitrosyl configuration at 1770 cm^−1^ disappears while the 1910 cm^−1^ GS band recovers. Finally, we have studied the thermal reversibility of the photo‐switching effect by re‐irradiating the sample with a 405 nm laser for 30 min and gradually heating it up to 300 K. Upon heating, spectra were collected at various temperatures (SI). The *v*(NO) band at 1770 cm^−1^ disappears at 170 K and indicates a thermal decay of the MSI state in the expected temperature range.[^34^, ^37^] Most remarkably, all three complexes ***1***, ***2***, ***3*** display similar light‐induced isomeric properties (SI).

In conclusion, Fe_*NO*_CuLnCo complexes constitute a new class of tuneable hetero‐tetra‐metallic species exhibiting dual properties. They have been obtained through a supramolecular approach by assembling functional building blocks: the nitroprusside Fe_*NO*_ for its photo‐switching properties and the CuLnCo trinuclear complexes for their magnetic properties. The isotropic Gd^III^ species (***1***) is a high spin molecule whereas Tb^III^ (***2***) and Dy^III^ (***3***) species present an SMM and a FI‐SMM behaviour, respectively. All three compounds exhibit thermally and optically reversible photo‐isomerism. Fe_*NO*_CuTbCo complex ***2*** is the finest example of the series as it is the first photo‐isomerisable hetero‐tetra‐metallic single‐molecule magnet ever reported in the literature. Given the nature of the photo‐switching process where the isomerisation of nitrosyl group does not imply any spin change in the iron(II) ion, no synergistic effects were expected. This was confirmed by EPR spectroscopy under irradiation (SI). We acknowledge that synergy has not been achieved in the present study. However, ongoing efforts are directed toward this objective, with a particular focus on octacyanidometallate precursors. The promising results are under investigation and will be published in the near future.

## Conflict of Interests

The authors declare no conflict of interest.

## Supporting information

As a service to our authors and readers, this journal provides supporting information supplied by the authors. Such materials are peer reviewed and may be re‐organized for online delivery, but are not copy‐edited or typeset. Technical support issues arising from supporting information (other than missing files) should be addressed to the authors.

Supporting Information
