# Supplementary material for: Unlocking New Frontiers: Photo‐Isomerism and Magnetic Properties in Multifunctional Hetero‐Tetra‐Metallic Complexes
Source: Chemistry. 2025 Feb 25;31(21):e202402601. doi: 10.1002/chem.202402601 (PMC11979684; doi:10.1002/chem.202402601)
Supplement: Supplementary file 1 — Supporting Information [file CHEM-31-e202402601-s001.pdf]

Chemistry–A European Journal

Supporting Information

Unlocking New Frontiers: Photo-Isomerism and Magnetic Properties in Multifunctional Hetero-Tetra-Metallic Complexes

Ingrid Suzana,* Jérémy Forté, Sébastien Pillet, El-Eulmi Bendeif, Moritz Malischewski,* and Valérie Marvaud*

Supporting information - Unlocking New Frontiers: Photo-Isomerism and Magnetic Properties in Multifunctional Hetero-Tetra-Metallic Complexes

Ingrid Suzana,*[a] Jeremy Forté,[a] Sebastien Pillet,[b] El-Eulmi Bendeif,[b] Moritz Malischewski,*[c] Valérie Marvaud*[a]

[a] Dr. I. Suzana, Dr. V. Marvaud, J. Forté
IPCM-CNRS, UMR 8232
Sorbonne Université
4 Place Jussieu, 75252 Paris Cedex 05 (France)
E-mail: igdsuzana@gmail.com, valerie.marvaud@sorbonne-universite.fr

[b] Dr. S. Pillet, Dr. E.-E. Bendeif
CRM2-CNRS
Université de Lorraine
54506 Vandoeuvre-les-Nancy (France)

[c] Dr. M. Malischewski
Institut für Chemie und Biochemie – Anorganische Chemie
Freie Universität Berlin
Fabeckstraße 34-36, 14195 Berlin, Deustchland
E-mail: moritz.malischewski@fu-berlin.de

Table of contents

1. Experimental part 2
- 1.1. Inductively Coupled Plasma Optical Emission Spectroscopy (ICP-OES) 2
- 1.2. Fourier-Transform InfraRed spectroscopy 2
- 1.3. Single crystal X-Ray Diffraction (SCXRD) 2
- 1.4. Magnetic measurements 2

2. Synthetic procedures 3
- 2.1. Synthesis of the dinuclear CuLn complexes 3
- 2.2. Synthesis of Kläui ligand 4
- 2.3. Synthesis of Fe_{NO}CuLnCo complexes procedure (A) 5
- 2.4. Synthesis of Fe_{NO}CuLnCo complexes procedure (B) 6

3. Structural characterisations 7
- 3.1. InfraRed Spectroscopy 7
- 3.2. Inductively Coupled Plasma Atomic Emission Spectroscopy (ICP-OES) 8
- 3.3. X-ray studies 9

4. Magnetic Studies 17
- 4.1. Static magnetic investigations of Fe_{NO}CuLnCo complexes 17
- 4.2. Dynamic magnetic investigations of Fe_{NO}CuLnCo complexes 19

5. Photo-switching studies 22
- 5.1. Gadolinium complex Fe_{NO}CuGdCo 23
- 5.2. Terbium complex Fe_{NO}CuTbCo 24
- 5.3. Dysprosium complexes Fe_{NO}CuDyCo 25
- 5.4. EPR measurements under irradiation 25

6. Literature 26

1. Experimental part

1.1. Inductively Coupled Plasma Optical Emission Spectroscopy (ICP-OES)

ICP-OES titrations were performed on an ICP-OES Spectrogreen Spectrometer at the Institut de PHYSIQUE du Globe de Paris (IPGP).

1.2. Fourier-Transform InfraRed spectroscopy

Fourier-Transform InfraRed (FT-IR) spectra were recorded as KBr pellets on a Bio-Rad Win-IR FTS 165 spectrometer (250-4000 cm^{-1}, 4 cm^{-1} resolution). The samples were mixed with KBr, finely ground and pressed to pellets. Band intensities are described as strong (s), medium (m), or weak (w).

FT-IR spectra at low temperature under irradiation were collected using a Nicolet 5700 FTIR spectrometer with a resolution of 2 cm^{-1} equipped with a Helium cryostat. The samples were finely ground, mixed with KBr, and pressed to pellets. To ensure good thermal contact for the low-temperature measurements, the pellets were contacted with silver paste on the cold finger of the cryostat. The samples were cooled to 10 K in the evacuated sample chamber (10^{-6} mbar). The cryostat is equipped with CsI windows allowing for collection of spectra in the range 4000-400cm^{-1} and for *in situ* irradiation with lasers. The excitation of the samples at low temperature was performed with different laser sources (405 nm and 1064 nm).

1.3. Single crystal X-Ray Diffraction (SCXRD)

The crystallographic data for all compounds were collected at 200 K from single crystals mounted on a loop fibber using a Bruker Kappa APEX-II CCD diffractometer using a micro-focused Cu-Kα radiation (λ = 1.54178 Å) or using a graphite-monochromated Mo-Kα radiation (λ = 0.71073 Å). Data collection was performed with Bruker APEX-III suite.[1] The data were reduced using SAINT program and SADABS for scaling and multi-scan absorption corrections.[2,3] Using Olex2 software package,[4] the structures were solved with ShelXT (Sheldrick, 2018) structure solution program using intrinsic phasing and refined with the ShelXL (Sheldrick, 2015) refinement package using full-matrix least squares methods.[5,6] All non-hydrogen atoms have been refined anisotropically. The structures were deposited at the Cambridge Crystallographic Data Centre with numbers CCDC 2350759-2350764.

1.4. Magnetic measurements

Static magnetic investigations (magnetic susceptibility, magnetisation) were obtained in the laboratory on a Superconducting QUantum Interference Device (SQUID): Quantum Design MPMS-XL magnetometer equipped with a 7 T magnet over the range of 2.1-300 K. The powdered samples (10±5 mg) were placed in a diamagnetic sample holder and the measurements realised in a 1 kOe applied field using the extraction technique. Raw data were corrected for the intrinsic diamagnetic contribution of the sample, estimated by Pascal's constants.

Dynamic magnetic investigations were obtained in the laboratory using a Quantum Design PPMS (Physical Property Measurement System), equipped with an alternative current (ac) susceptometer insert over the range of 10-10k Hz, 2.1-10 K and 0-2 kOe dc field.

EPR measurements were performed with a Bruker ESP 300 spectrometer, at X Band frequency (9.5 GHz) and a 4-300 K variable temperature cryostat under irradiation. The magnetic field modulation frequency was set at 100k Hz and the modulation amplitude and the microwave power were both adjusted to avoid saturation. The spectra were measured at temperatures between 4 K and 300 K. The excitation of the samples at low temperature was performed with laser sources at 405 and 488 nm.

2. Synthetic procedures

All chemicals were purchased from commercial suppliers and used without further purification.

2.1. Synthesis of the dinuclear CuLn complexes

Valen ligand – N,N'-bis(3-methoxy-salicylidene)ethylenediamine

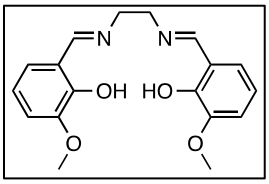

O-vanillin (16 g, 108 mmol) was dissolved in methanol (100 mL) and the ethylenediamine (3.245 g, 54 mmol) was slowly added to the stirring yellow solution. Once the addition completed, a yellow solid precipitated out and the resulting mixture was stirred at 60°C for 30 min. Then, it was cooled down to 0°C and the bright yellow solid was filtered out. It was then washed with methanol and dried with diethyl ether to yield 91% of product.

$C_{18}H_{18}N_2O_4$, Yield; 91%. Selected IR data (FT-IR, KBr pellets): σ (cm^{-1}) 1633 (s), 1470 (s), 1410 (m), 1252 (s), 1170(w), 1080 (m), 961 (m), 837 (w), 791 (m), 740 (m), 620 (w). ^1H NMR ($CDCl_3$) : δ (ppm) 13.56 (s, 2H, OH), 8.31 (s, 2H, C=N-H), 6.85 (dd, J = 1.7 Hz, 2H), 6.78 (t, J=7.7 Hz, 2H), 3.89 (s, 6H), 6.90

Cu-valen complex – [Cu(N,N'-bis(3-methoxy-salicylidene)ethylenediamine)]

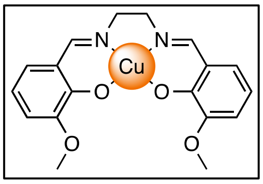

Valen ligand (3.28 g, 10 mmol, 1 equiv.) was suspended in 60 mL of methanol. To the stirring solution, $Cu(OAc)_2{\cdot}xH_2O$ (2.03 g, 10 mmol, 1 equiv.) in water (20 mL) was added and the resulting green solution was stirred at 60°C for 1 h. Then, the mixture was cooled down to 0°C and the solid was filtered out. It was washed with a minimal amount of methanol, acetone and diethyl ether.

Yield; 88%. Selected IR data (FT-IR, KBr pellets): **σ** (cm^{-1}) 1643 (s), 1603 (m), 1546 (w), 1474 (m), 1444 (m), 1321 (w), 1242 (s), 1220 (s), 1169 (w), 1085 (m), 987 (w), 743 (w), 725 (m).

General Procedure for the synthesis of CuLn-valen complexes (CuLn) – Ln = Gd, Tb, Dy

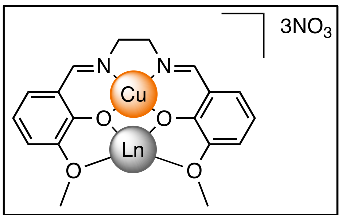

Cu-valen complex (3.92 g, 1 mmol, 1 equiv.) was suspended in acetone and stirred for 5 min. To the dark green suspension, a stoichiometric amount of the lanthanoid nitrate salt $Ln(NO_3)_3{\cdot}xH_2O$ (1 mmol, 1 equiv.) was added as a solid and stirred for about 30 min. The mixture was then filtered, and the brown solid washed with acetone and dried with diethyl ether.

$[Cu(C_{18}H_{18}N_2O_4)Gd](NO_3)_3{\cdot}Me_2CO$ **CuGd**

$Gd(NO_3)_3{\cdot}xH_2O$ mass; 451 mg. Yield; 88%. Selected IR data (FT-IR, KBr pellets): **σ** (cm^{-1}) 1637 (m), 1558 (w), 1472 (m),1457 (m), 1384 (s), 1241 (w), 1223 (m), 1078 (w), 953 (w).

$[Cu(C_{18}H_{18}N_2O_4)Tb](NO_3)_3{\cdot}Me_2CO$ **CuTb**

$Tb(NO_3)_3{\cdot}xH_2O$ mass; 453 mg. Yield; 91%. Selected IR data (FT-IR, KBr pellets): **σ** (cm^{-1}) 1638 (m), 1558 (w), 1472 (m), 1458 (m), 1385 (s), 1242 (w), 1223 (m), 1078 (w), 952 (w).

$[Cu(C_{18}H_{18}N_2O_4)Dy](NO_3)_3{\cdot}Me_2CO$ **CuDy**

$Dy(NO_3)_3{\cdot}xH_2O$ mass; 457 mg. Yield; 90%. Selected FT-IR data (FT-IR, KBr pellets): **σ** (cm^{-1}) 1635 (m), 1558 (w), 1472 (m), 1458 (m), 1384 (s), 1241 (w), 1222 (m), 1076 (w), 951 (w).

2.2. Synthesis of Kläui ligand

Bis[(cyclopentadienyl)tris(dimethylphosphito-P)Cobalt(III)-O,O',O"]Cobalt(II)

Cobaltocene Cp_2Co (1.8 g, 9.55 mmol) was heated with 4.5 mL of distilled dimethylphosphite $HP(O)(OCH_3)_2$ (49.05 mmol) under reflux conditions (90°C) and inert atmosphere overnight. It yielded a dark orange mixture; the excess of $HP(O)(OCH_3)_2$ was filtered off and the product was washed with ethanol and hexane to afford 2.561 g of an orange solid in 81% yield.

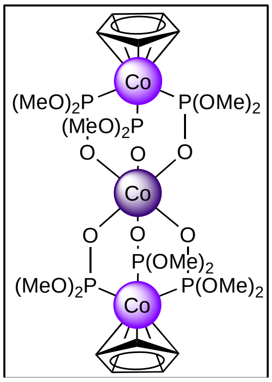

$[Co(CpCo(PO(OMe)_2)_3)_2]$ complex

Yield; 81%. Selected IR data (ATR): σ (cm^{-1}) 3312 (w), 2941 (w), 1461 (w), 1424 (w), 1125 (m), 1115 (m), 1032 (s), 1000 (s), 835 (m), 758 (s), 716 (s).

Kläui ligand – Sodium cyclopentadieyltris(dimethylphosphito-P)cobalate(III)

$[Co(CpCo(PO(OMe)_2)_3)_2]$ (2.561 g, 3 mmol) was dissolved in 30 mL of methanol whereupon NaCN (1.135 g, 23.2 mmol) was added in small portions at 0°C.[1] After an hour under constant air flux, the dark orange solution was stirred under ambient conditions overnight to afford a bright orange solution. The solvent was reduced and the dry solid extracted with CH_2Cl_2 by means of a Soxhlet extractor. Evaporation of the solvent yielded 0.712 g of yellow crystals (78%).

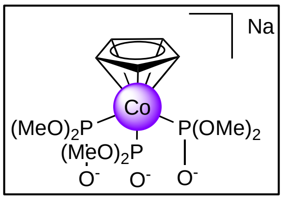

$[CpCo(PO(OMe)_2)_3]Na$ **Co-Kläui**

Yield; 78%. Selected IR data (ATR): σ (cm^{-1}) 3099 (w), 2943 (w), 1461 (w), 1428 (w), 1168 (m), 1035 (m), 991 (s), 834 (m), 747 (s), 698 (s) ^1H NMR (400MHz, $CDCl_3$): δ (ppm) 5.03 (s, 1H, C_5H_5), 3.59 (virt.q, 18H, OCH_3).

2.3. Synthesis of the trinuclear CuLnCo precursors

General Procedure for the synthesis of CuLnCo-valen complexes (CuLnCo) – Ln = Gd, Tb, Dy

In a beaker, CuLn (20.1 mg, 0.025 mmol, 1.25 equiv.) was dissolved in 15 mL of MeOH whereupon Kläui ligand (9.5 mg, 0.02mmol, 1equiv.) was added as a solid over a few minutes. Once the dissolution is complete, sodium perchlorates salts $NaClO_4$ (15.3 mg, 0.125 mmol, 6.25 equiv.) were added to the purple solution.[2] Finally, the solution was left for slow evaporation to yield crystals. Note that evaporation may lead to crystallisation of two types of crystals: the Cu-valen as green platelets and $[CuLnCo\text{-}v](ClO_4)_2$ as red platelets. They can be easily separated as the CuLnCo-v crystals are soluble in acetone in which they crystallise with Et_2O vapours.

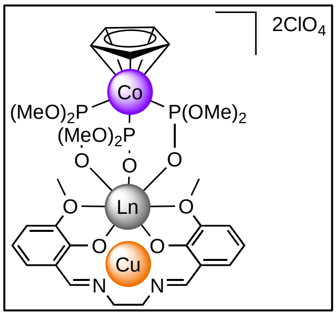

$[Cu(C_{18}H_{18}N_2O_4)Gd(CpCo(PO(OMe)_2)_3)(H_2O)](ClO_4)_2{\cdot}5H_2O$, **CuGdCo**

CuGd mass; 19.8mg. Yield; 32%. Crystals were isolated as red platelets. Selected FT-IR data (FT-IR, KBr pellets): σ (cm^{-1}) 1633 (s), 1607 (m), 1474 (m), 1456 (m), 1295 (s), 1246 (w), 1224 (m), 1122 (s), 1082 (s), 1039 (s), 1005 (s), 779 (m), 731 (m), 624 (m), 588 (m).

[1] NaCN is toxic and mutagenic.
[2] Sodium perchlorates salts $NaClO_4$ are potentially explosive.

[Cu($C_{18}H_{18}N_2O_4$)Tb(CpCo(PO(OMe)$_2$)$_3$)(H_2O)](ClO_4)$_2$·6H_2O, **CuTbCo**

CuTb mass; 19.8 mg Yield; 34%. Crystals were isolated as red platelets. Selected FT-IR data (FT-IR, KBr pellets): σ (cm^{-1}) 1632 (s), 1607 (m), 1474 (m), 1456 (m), 1297 (s), 1241 (w), 1224 (m), 1121 (s), 1083 (s), 1036 (s), 1004 (s), 779 (m), 729 (m), 625 (m), 589 (m).

[Cu($C_{18}H_{18}N_2O_4$)Dy(CpCo(PO(OMe)$_2$)$_3$)(H_2O)](ClO_4)$_2$·6H_2O, **CuDyCo**

CuDy mass; 19.8 mg Yield; 27%. Crystals were isolated as red platelets. Selected FT-IR data (FT-IR, KBr pellets): σ (cm^{-1}) 1633 (s), 1607 (m), 1474 (m), 1456 (m), 1296 (s), 1241 (w), 1225 (m), 1121 (s), 1082 (s), 1032 (s), 1002 (s), 779 (m), 731 (m), 625 (m), 588 (m).

2.4. Synthesis of Fe$_{NO}$CuLnCo complexes procedure (A)

General Procedure (A) for the synthesis of Fe$_{NO}$CuLnCo complexes – Ln = Gd, Tb, Dy

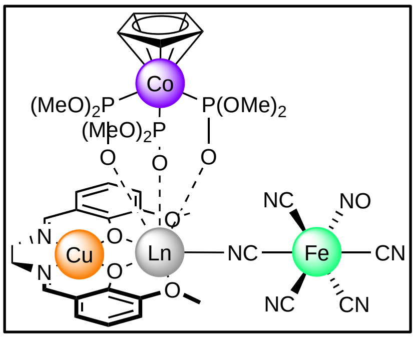

In a beaker, CuLn-ligand (19.8 mg, 0.025 mmol, 1.25 equiv.) was dissolved in 15 mL of MeCN/H_2O (3:1) whereupon Kläui ligand (0.020 mmol, 1 equiv.) was added as a powder over several minutes, yielding the formation of the CuLnCo$^+$ building block *in situ*. Then, nitroprusside salts Na_2[Fe(NO(CN)$_5$]·2H_2O (7.5 mg, 0.025 mmol, 1.25 equiv.) in 2 mL of water were added to the green solution. After some minutes stirring, the resulting solution was left for slow evaporation to yield green crystals. They were collected by filtration, washed with a minimal amount of MeCN/H_2O (3:1) and dried in air. Samples for crystallography were maintained in contact with the mother liquor to preserve the optimum quality of the crystalline samples.

[Cu($C_{18}H_{18}N_2O_4$)Gd(CpCo(PO(OMe)$_2$)$_3$)(μ-CN)Fe(CN)$_4$NO]·6H_2O **Fe$_{NO}$CuGdCo (*1*)**

Crystals were isolated as red prisms. Yield; 83% Selected FT-IR data (FT-IR, KBr pellets): σ (cm^{-1}) 2180 (w), 2157 (w), 2138 (m), 1905 (s), 1626 (s), 1474 (m), 1454(m), 1300 (m), 1247 (m), 1224 (m), 1125 (s), 1081 (m), 1038 (s), 1009 (m), 857 (w), 779 (m), 730 (m), 586 (w).

[Cu($C_{18}H_{18}N_2O_4$)Tb(CpCo(PO(OMe)$_2$)$_3$)(μ-CN)Fe(CN)$_4$NO]·7H_2O **Fe$_{NO}$CuTbCo (*2*)**

Crystals were isolated as red prisms. Yield; 80% Selected FT-IR data (FT-IR, KBr pellets): σ (cm^{-1}) 2187 (w), 2138 (m), 1906 (s), 1627 (s), 1474 (m), 1299 (m), 1248 (m), 1224 (m), 1118 (s), 1081 (m), 1038 (s), 1009 (m), 857 (w), 779 (m), 730 (m), 587 (w).

[Cu($C_{18}H_{18}N_2O_4$)Dy(CpCo(PO(OMe)$_2$)$_3$)(μ-CN)Fe(CN)$_4$NO]·6H_2O **Fe$_{NO}$CuDyCo (*3*)**

Crystals were isolated as red prisms. Yield; 76% Selected FT-IR data (FT-IR, KBr pellets): σ (cm^{-1}) 2173 (w), 2140 (m), 1907 (s), 1621 (s), 1472 (m), 1299 (m), 1248 (m), 1224 (m), 1118 (s), 1081 (m), 1038 (s), 1008 (m), 857 (w), 779 (m), 730 (m), 589 (w).

2.5. Synthesis of Fe_{NO}CuLnCo complexes procedure (B)

General Procedure (B) for the synthesis of Fe_{NO}CuLnCo complexes – Ln = Gd, Tb, Dy

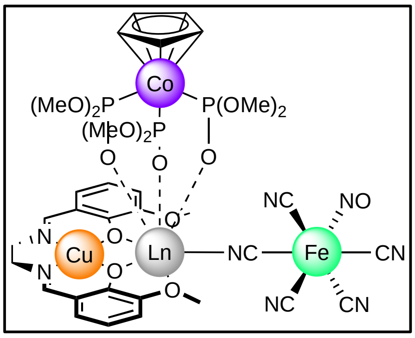

In a beaker, CuLn (19.8 mg, 0.025 mmol, 1.25equiv.) was dissolved in 15 mL of MeCN/H_2O whereupon Kläui ligand (0.020mmol, 1equiv.) was added as a powder over several minutes, yielding the formation of the CuLnCo building block *in situ*. Then, nitroprusside salts $Na_2[Fe(NO(CN)_5]\cdot 2H_2O$ (7.5 mg, 0.075 mmol, 1.25 equiv.) in 2 mL of water were added to the purple solution. Finally, tetraphenyl arsonium chloride $[AsPh_4]Cl$[3] (41.1 mg, 0.125 mmol, 6.25 equiv.) were added and the resulting solution was left for slow evaporation to yield crystals. They were collected by filtration, washed with a mixture of MeCN/H_2O (3:1) and dried in air. Samples for crystallography were maintained in contact with the mother liquor to preserve the optimum quality of the crystalline samples.

$[Cu(C_{18}H_{18}N_2O_4)Gd(CpCo(PO(OMe)_2)_3)(\mu\text{-}CN)Fe(CN)_4NO]\cdot 6H_2O$ **Fe_{NO}CuGdCo (*1*)**

Crystals were isolated as red prisms. Yield; 82 % ICP-OES Found: Fe, 4.44; Cu, 4.86, Gd, 11.45, Co, 4.51 Calc for $C_{34}H_{55}N_8O_{21}P_3$FeCuCoGd: Fe, 4.17; Cu, 4.74; Co, 4.40; Gd, 11.73 %. Selected FT-IR data (FT-IR, KBr pellets): σ (cm^{-1}) 2180 (m), 2157 (w), 2138 (m), 1905 (s), 1626 (s), 1606 (m), 1474 (m), 1454 (m), 1300 (m), 1247 (m), 1224 (m), 1125 (s), 1081 (m), 1038 (s), 1009 (s), 857 (w), 779 (m), 731 (m), 586 (w).

$[Cu(C_{18}H_{18}N_2O_4)Tb(CpCo(PO(OMe)_2)_3)(\mu\text{-}CN)Fe(CN)_4NO]\cdot 7H_2O$ **Fe_{NO}CuTbCo (*2*)**

Crystals were isolated as red prisms. Yield; 83 % ICP-OES Found: Fe, 4.25; Cu, 4.84; Co, 4.82; Tb, 11.71 Calc. for $C_{34}H_{55}N_8O_{21}P_3$FeCuCoTb: Fe, 4.16; Cu, 4.74; Co, 4.39; Tb, 11.84 %. Calc for FeCuTbCo. Selected FT-IR data (FT-IR, KBr pellets): σ (cm^{-1}) 2187 (w), 2138 (m), 1906 (s), 1627 (s), 1474 (m), 1299 (m), 1248 (m), 1224 (m), 1118 (s), 1081 (m), 1038 (s), 1009 (m), 857 (w), 779 (m), 730 (m), 587 (w).

$[Cu(C_{18}H_{18}N_2O_4)Dy(CpCo(PO(OMe)_2)_3)(\mu\text{-}CN)Fe(CN)_4NO]\cdot 6H_2O$ **Fe_{NO}CuDyCo (*3*)**

Crystals were isolated as red prisms. Yield; 79 % ICP-OES titration Found: Fe, 4.35; Cu, 4.90; Co, 4.75; Dy, 11.81 % Calc. for $C_{34}H_{55}N_8O_{21}P_3$FeCuCoDy: Fe, 4.15; Cu, 4.72; Co, 4.38; Dy, 12.08 % Selected FT-IR data (FT-IR, KBr pellets): σ (cm^{-1}) 2185 (w), 2138 (m), 1905 (s), 1626 (s), 1474 (m), 1299 (m), 1248 (m), 1224 (m), 1118 (s), 1081 (m), 1038 (s), 1008 (m), 857 (w), 779 (m), 730 (m), 587 (w).

[3] Tetraphenyl arsonium chloride salts $[AsPh_4]Cl$ are toxic.

3. Structural characterisations

3.1. InfraRed Spectroscopy

3.1.1. InfraRed spectra of valen complexes CuLnCo

InfraRed spectra of CuLnCo (Ln = Gd, Tb, Dy) are similar – hereafter is [CuGdCo](ClO_4)$_2$ spectrum.

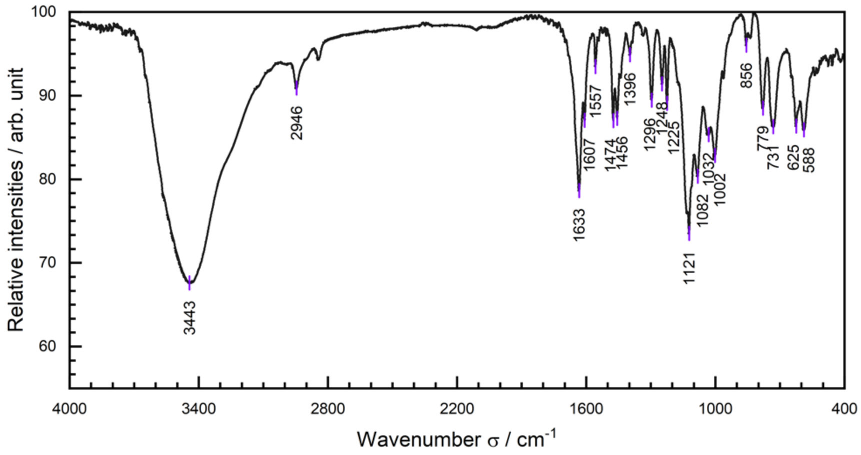

Figure S1. InfraRed spectrum of [CuTbCo](ClO_4)$_2$.

3.1.2. Nitroprusside precursor Fe$_{NO}$

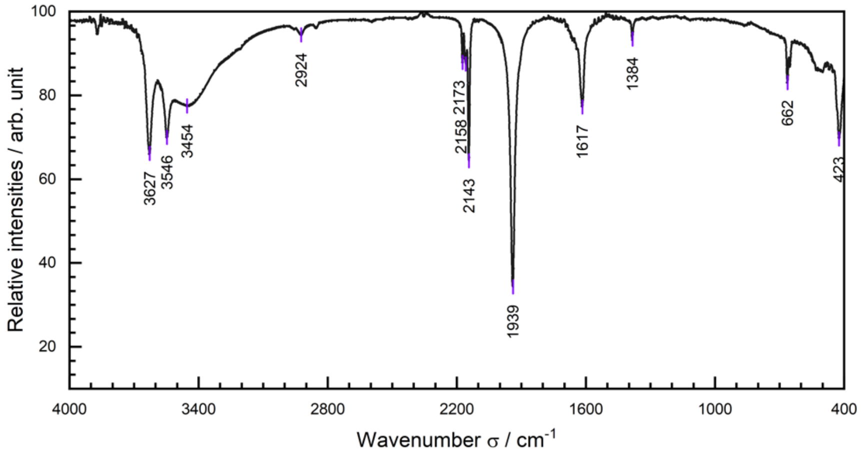

Figure S2. InfraRed spectrum of sodium nitroprusside $Na_2[FeNO(CN)_5]$.

3.1.3. Fe$_{NO}$CuLnCo complexes

Table S1. Selected peaks of InfraRed spectrum of Fe$_{NO}$CuGdCo

	**Fe$_{NO}$CuLnCo**		
ν	Fe$_{NO}$CuGdCo (***1***)	Fe$_{NO}$CuTbCo (***2***)	Fe$_{NO}$CuDyCo (***3***)
νC≡N, m	2180	2187	2185
νC=N, m	2157<br>2138	2138	2138
νN=O, s	1905	1906	1905
νC=N, s	1626<br>1606	1627	1626
νC–H, s	1474	1474	1474

	**Fe$_{NO}$CuLnCo**		
ν	Fe$_{NO}$CuGdCo (***1***)	Fe$_{NO}$CuTbCo (***2***)	Fe$_{NO}$CuDyCo (***3***)
νC-H, s	1009	1009	1008
νP=O, s	586	587	587

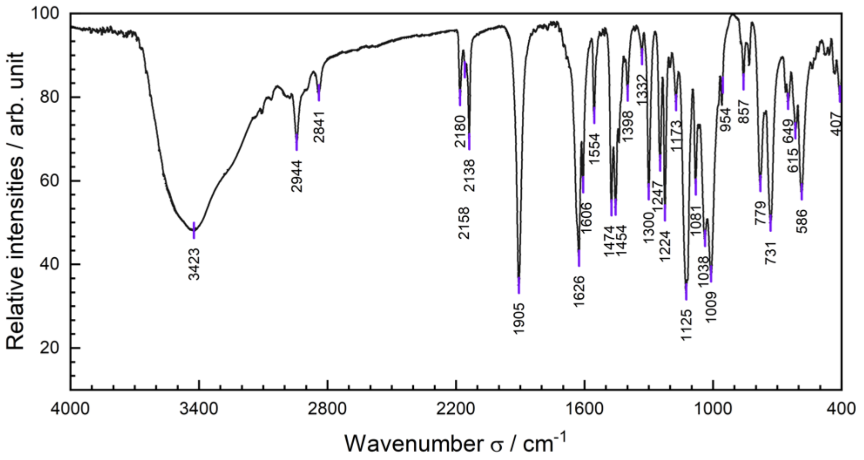

Figure S3. InfraRed spectrum of sodium nitroprusside $Na_2[FeNO(CN)_5]$.

3.2. Inductively Coupled Plasma Atomic Emission Spectroscopy (ICP-OES)

Table S2. Elemental analysis of Fe$_{NO}$CuLnCo compounds by ICP-OES spectroscopy

	**Fe$_{NO}$CuLnCo**		
	Mass concentration (%)		
	Fe$_{NO}$CuGdCo (***1***)	Fe$_{NO}$CuTbCo (***2***)	Fe$_{NO}$CuDyCo (***3***)
Fe exp	4.44	4.25	4.35
Fe theo	*4.17*	*4.16*	*4.15*
Co exp	4.51	4.82	4.75
Co theo	*4.40*	*4.39*	*4.38*
Cu exp	4.86	4.84	4.90
Cu theo	*4.74*	*4.74*	*4.72*
Ln exp	11.45	11.71	11.81
Ln theo	*11.73*	*11.84*	*12.08*

3.3. X-ray studies

3.3.1. Crystallographic tables of CuLnCo – Ln = Gd, Tb, Dy

Table S3. Crystallographic data for compounds [CuLnCo](ClO_4)$_2$ – Ln = Gd, Tb, Dy

	**[CuGdCo-v](ClO_4)$_2$**	**[CuTbCo-v](ClO_4)$_2$**	**[CuDyCo-v](ClO_4)$_2$**
**CCDC**	2350760	2350759	2350761
Empirical formula	$C_{29}H_{55}Cl_2CoCuN_2O_{28}P_3Gd$	$C_{29}H_{55}Cl_2CoCuN_2O_{28}P_3Tb$	$C_{29}H_{55}Cl_2CoCuN_2O_{28}P_3Dy$
Moiety Formula	$C_{29}H_{45}CoCuN_2O_{15}P_3Gd^{2+}$, 2($ClO_4^-$), 5($H_2O$)	$C_{29}H_{45}CoCuN_2O_{15}P_3Tb^{2+}$, 2($ClO_4^-$), 5($H_2O$)	$C_{29}H_{45}CoCuN_2O_{15}P_3Dy^{2+}$, 2($ClO_4^-$), 5($H_2O$)
Formula weight	1323.28	1324.95	1328.52
Temperature / K	200	200	200
Crystal system	triclinic	triclinic	triclinic
Space group	P-1	P-1	P-1
a / Å	10.8029(4)	10.7781(4)	10.7713(19)
b / Å	12.8410(5)	12.8210(5)	12.833(2)
c / Å	19.2274(7)	19.2733(8)	19.309(3)
α / °	71.301(2)	71.494(2)	71.388(2)
β / °	74.489(2)	74.242(2)	74.190(3)
γ / °	74.602(2)	74.483(2)	74.420(2)
V / Å^3	2386.73(16)	2382.21(17)	2385.1(7)
Z	2	2	2
ρ_{calc} / g.cm^{-1}	1.841	1.847	1.850
μ / mm^{-1}	14.873	2.56	2.641
F(000)	1332	1334	1336
Crystal size / mm^3	0.15 × 0.09 × 0.06	0.46 × 0.26 × 0.12	0.3 × 0.2 × 0.1
Radiation	CuKα (λ = 1.54178)	MoKα (λ = 0.71073)	MoKα (λ = 0.71073)
2θ range for data collection / °	4.952 to 133.486	2.276 to 61.272	2.274 to 50.498
Index ranges	-12 ≤ h ≤ 12, -15 ≤ k ≤ 15, -19 ≤ l ≤ 22	-15 ≤ h ≤ 15, -18 ≤ k ≤ 18, -27 ≤ l ≤ 27	-10 ≤ h ≤ 12, -15 ≤ k ≤ 15, -23 ≤ l ≤ 16
Reflections collected	36188	141170	10850
Independent reflections	8445 [R_{int} = 0.0640, R_{sigma} = 0.0490]	14650 [R_{int} = 0.0522, R_{sigma} = 0.0349]	8032 [R_{int} = 0.0362, R_{sigma} = 0.1006]
Data/restraints/parameters	8445/55/707	14650/0/721	8032/544/718
Goodness-of-fit on F^2	1.008	1.165	0.982
Final R indexes [$I \leq 2\sigma \leq (I)$]	R_1 = 0.0381, wR_2 = 0.0908	R_1 = 0.0403, wR_2 = 0.0826	R1 = 0.0836, wR2 = 0.1028
Final R indexes [all data]	R_1 = 0.0462, wR_2 = 0.0945	R_1 = 0.0556, wR_2 = 0.0876	R1 = 0.0501, wR2 = 0.0934
Largest diff. peak/hole / eÅ^{-3}	0.80/-0.65	1.53/-1.34	1.22/-0.76

3.3.2. Crystallographic tables of Fe_{NO}CuLnCo – Ln = Gd, Tb, Dy

Table S4. Crystallographic data for compounds Fe_{NO}CuLnCo – Ln = Gd, Tb, Dy

	**Fe_{NO}CuGdCo (*1*)**	**Fe_{NO}CuTbCo (*2*)**	**Fe_{NO}CuDyCo (*3*)**
**CCDC**	2350763	2350762	2350764
Empirical formula	$C_{34}H_{55}CoCuFeGdN_8O_{21}P_3$	$C_{34}H_{55}CoCuFeN_8O_{21}P_3Tb$	$C_{34}H_{55}CoCuDyFeN_8O_{21}P_3$
Moiety formula	$C_{34}H_{43}CoCuFeN_8O_{15}P_3Gd$, 6($H_2O$)	$C_{34}H_{43}CoCuFeN_8O_{15}P_3Tb$, 6($H_2O$)	$C_{34}H_{43}CoCuFeN_8O_{15}P_3Dy$, 6($H_2O$)
Formula weight	1340.34	1342.01	1345.59
Temperature / K	200	200	200
Crystal system	triclinic	triclinic	triclinic
Space group	P-1	P-1	P-1
a / Å	10.9090(5)	10.9253(13)	10.9098(3)
b / Å	14.7616(7)	14.7614(17)	14.7343(4)
c / Å	17.9091(7)	17.911(2)	17.8811(5)
α / °	73.611(3)	73.561(2)	73.6680(10)
β / °	74.955(3)	75.065(2)	75.1300(10)
γ / °	68.734(3)	68.767(2)	68.9420(10)
V / $Å^3$	2538.2(2)	2543.1(5)	2535.01(12)
Z	2	2	2
ρ_{calc} / $g.cm^{-1}$	1.754	1.753	1.763
μ / mm^{-1}	15.158	2.562	14.601
F(000)	1348	1350	1352
Crystal size / mm^3	0.25 × 0.15 × 0.1	0.388 × 0.244 × 0.231	0.19 × 0.11 × 0.1
Radiation	CuKα (λ = 1.54178)	MoKα (λ = 0.71073)	CuKα (λ = 1.54178)
2θ range for data collection / °	5.224 to 133.504	2.408 to 61.244	8.82 to 133.31
Index ranges	-12 ≤ h ≤ 12, -17 ≤ k ≤ 17, -21 ≤ l ≤ 19	-15 ≤ h ≤ 15, -21 ≤ k ≤ 21, -25 ≤ l ≤ 25	-12 ≤ h ≤ 12, -17 ≤ k ≤ 17, -21 ≤ l ≤ 21
Reflections collected	41710	126552	38079
Independent reflections	8966 [R_{int} = 0.0738, R_{sigma} = 0.0578]	15620 [R_{int} = 0.0277, R_{sigma} = 0.0157]	8950 [R_{int} = 0.0407, R_{sigma} = 0.0321]
Data/restraints/parameters	8966/0/632	15620/211/705	8950/0/639
Goodness-of-fit on F^2	1.068	1.037	1.032
Final R indexes [$I \leq 2\sigma \leq (I)$]	R_1 = 0.0599, wR_2 = 0.1431	R_1 = 0.0237, wR_2 = 0.0632	R_1 = 0.0311, wR_2 = 0.0797
Final R indexes [all data]	R_1 = 0.0775, wR_2 = 0.1542	R_1 = 0.0294, wR_2 = 0.0666	R_1 = 0.0358, wR_2 = 0.0822
Largest diff. peak/hole / $eÅ^{-3}$	1.99/-0.89	0.88/-1.25	0.61/-0.65

3.3.3. Description of Fe_{NO}CuGdCo (*1*)

Regarding the nitroprusside anion, the iron(II) atom is coordinated to five cyanides CN^- and one nitrosyl NO^+ group. As expected from the literature, Fe^{II} binds to the Gd^{III} ion through a cyanide bridging ligand in the cis-position of the NO^+ group.[7,8] It features various coordination bond length depending on the ligand nature: the Fe-N bond (1.72 Å) is considerably shorter than Fe-C bonds ranging from 1.881 Å to 1.949 Å for the bridging cyanide. All these differences in bonds around the iron(II) ion are in line with the literature and result in a pseudo octahedral geometry.[7–11] Longest CN bonds are that of the bridging group and the one being in the trans-position of the NO^+ group. These account for a shared electronic density between the two metal ions and a less efficient π-back donation on the cyanide ligand in the trans-position of the nitrosyl group.[12] All CN group are bent and especially the ones in cis-position of the bridging cyanide and the NO^+ group. In comparison, the NO^+ ligand is quasi linear with a Fe-N-O angle value of 179.0°.

The copper(II) ion is found in the N_2O_2 cavity of the valen ligand where it adopts a square planar geometry. The N1-Cu1-N2 and O3-Cu1-O2 angles are rather closed with a value of 85.20° whereas the N1-Cu1-O2 and N2-Cu1-O3 are more open with a value of 94.75°. The average value of the coordination bonds is 1.92 Å with bond lengths ranging from 1.906 Å to 1.928 Å. The closest donor atom to the copper(II) is O6 from the Kläui O_3 donor set (3.314 Å).

In the O_2O_2 cavity of the compartmental ligand lies the gadolinium(III) ion where it binds to the oxygen ions of the methoxy and phenoxo groups. In addition, it is capped by the Kläui ligand O_3 donor set and binds to N3 that belongs to one of the nitroprusside cyanide ligands. Its coordination number reaches 9 owing to one water molecule completing its coordination sphere. The bond distances Gd1-O*methoxy* (2.78 Å) are distinctly longer than the Gd-N*cyanide* (2.50 Å), Gd-O*phenoxo* (2.40 Å), Gd-O*water* (2.44 Å), and Gd-O*kläui* (2.35 Å). Interestingly, the Ln-NC bridge is non-linear with an angle Ln-N-C value of 170.8(7)° and it is in line with the values reported in the literature for cyanide group bridging lanthanoids and nitroprusside (156.5-176.3°).[7,8] The lowest distortion parameter was found for a muffin shape with a C_s symmetry, with the basal trigonal and pentagonal planes are formed by O5, O6 and O7 atoms (Kläui ligand O_3 donor set) and the O1, O2, O3, O4, N3 atoms (valen O_2O_2 cavity and cyanide bridging ligand) and an O14 atom at the vertex of the muffin.

3.3.1. Key structural parameters in Fe*NO*CuLnCo

*Table S5. Selected bond lengths (in Å) and angles (in °) of compounds Fe_{NO}CuGdCo (**1**)*

**Fe*NO*CuGdCo (*1*)**			
Interatomic distances (Å)		Angles (°)	
Fe-C free	1.927(9)<br>1.93(1)<br>1.881(9)<br>1.91(1)	Fe-C-N free	176(1)<br>175.8(9)<br>174.7(9)<br>177.3(9)
Fe-C bridge	1.949(8)	Fe-C-N bridge	176.7(8)
Fe-N	1.72(1)	Fe-N-O	179.0(9)
Free CN	1.13(1)<br>1.13(1)<br>1.17(1)<br>1.15(1)	N1-Cu-N2	85.8(3)
Bridging CN	1.13(1)	N1-Cu-O2	94.7(3)
Gd-N3	2.498(7)	N2-Cu-O3	94.7(3)
Gd-O_{metoxy}	2.723(6)<br>2.841(6)	O2-Cu-O3	84.7(2)
Gd-$O_{phenoxo}$	2.393(7)<br>2.405(5)	Gd-N-C	170.91
Gd-$O_{kläui}$	2.326(7)<br>2.429(5)<br>2.306(5)	Gd-O3-Cu	99.85
Gd-O_{water}	2.440(5)	Gd-O2-Cu	100.06
Cu-N	1.918(9)<br>1.928(6)	GdO_2Cu (ϕ)	31.16
Cu-$O_{phenoxo}$	1.912(5)<br>1.905(7)		
Co-P	2.166(2)<br>2.168(3)<br>2.169(3)		
Co-C	2.078(9)<br>2.07(1)<br>2.07(1)<br>2.07(1)<br>2.07(1)		
Gd-Fe	5.567(1)		
Gd-Cu	3.314(1)		
Gd-Co	4.333(1)		
Gd1-Gd2	9.937(1)		
Cu1-Cu2	6.669(1)		
Gd1-Cu2	7.787(1)		

i : symmetry code (1-x, -y, 1-z)

*Table S6. Selected bond lengths (in Å) and angles (in °) of compounds Fe_{NO}CuTbCo (**2**)*

**Fe_{NO}CuTbCo (*2*)**			
Interatomic distances (Å)		Angles (°)	
Fe-C free	1.939(3)<br>1.939(2)<br>1.832(2)<br>1.897(3)	Fe-C-N free	176.99
Fe-C bridge	1.937(4)	Fe-C-N bridge	177.27
Fe-N	1.708(2)	Fe-N-O	178.99
Free CN	1.142(4)<br>1.145(3)<br>1.150(4)<br>1.144(4)	N1-Cu-N2	86.24
Bridging CN	1.139(4)	N1-Cu-O2	94.74
NO	1.146(3)	N2-Cu-O3	94.49
Tb-N3	2.485(2)	O2-Cu-O3	84.43
Tb-$O_{methoxy}$	2.852(2)<br>2.723(2)	Tb-N-C	171.99
Tb-$O_{phenoxo}$	2.380(2)<br>2.387(1)	Tb-O3-Cu	99.69
Tb-$O_{kläui}$	2.283(1)<br>2.298(2)<br>2.417(1)	Tb-O2-Cu	99.97
Tb-O_{water}	2.427(2)	TbO_2Cu (ϕ)	31.73
Cu-N	1.914(2)<br>1.921(2)		
Cu-$O_{phenoxo}$	1.905(2)<br>1.904(1)		
Co-P	2.1674(7)<br>2.1624(7)<br>2.1634(7)		
Co-C	2.13(1)<br>2.09(1)<br>2.07(1)<br>2.09(2)<br>2.12(2)		
Tb-Fe	5.554		
Tb-Cu	3.295		
Tb-Co	4.326		
Tb1-Tb2	9.901		
Cu1-Cu2	6.677		
Tb1-Cu2	7.775		

i : symmetry code (1-x, -y, 1-z)

*Table S7. Selected bond lengths (in Å) and angles (in °) of compounds Fe_{NO}CuDyCo (**3**)*

**Fe_{NO}CuDyCo (*3*)**			
Interatomic distances (Å)		Angles (°)	
Fe-C free	1.942(4)<br>1.938(4)<br>1.904(4)<br>1.900(4)	Fe-C-N free	175.9(4)<br>177.7(4)<br>177.5(4)<br>176.0(4)
Fe-C bridge	1.940(4)	Fe-C-N bridge	176.8(3)
Fe-N	1.705(4)	Fe-N-O	179.4(4)
Free CN	1.134(6)<br>1.135(6)<br>1.142(6)<br>1.144(5)	N1-Cu-N2	86.2(1)
Bridging CN	1.145	N1-Cu-O2	94.9(1)
NO	1.137(6)	N2-Cu-O3	94.5(1)
Dy-N3	2.467(3)	O2-Cu-O3	84.3(1)
Dy-$O_{methoxy}$	2.866(3)<br>2.719(2)	Dy-N-C	172.1(3)
Dy-$O_{phenoxo}$	2.364(3)<br>2.373(2)	Dy-O3-Cu	99.5(1)
Dy-$O_{kläui}$	2.275(2)<br>2.286(3)<br>2.412(2)	Dy-O2-Cu	99.8(1)
Dy-O_{water}	2.419(2)	DyO_2Cu (ϕ)	31.90
Cu-N	1.919(4)<br>1.929(3)		
Cu-$O_{phenoxo}$	1.906(3)<br>1.907(2)		
Co-P	2.167(1)<br>2.161(1)<br>2.163(1)		
Co-C	2.072(5)<br>2.073(5)<br>2.073(4)<br>2.080(5)<br>2.071(5)		
Ln-Fe	5.541		
Ln-Cu	3.279		
Ln-Co	4.309		
Dy1-Dy2	9.884		
Cu1-Cu2	6.691		
Dy1-Cu2	7.777		

i : symmetry code (1-x, -y, 1-z)

3.3.1.1. Packing analysis of Fe_{NO}CuGdCo (*1*)

All Fe_{NO}CuLnCo complexes are isostructural, only the crystal packing of ***1*** is detailed here as it is strictly identical to that of its analogues.

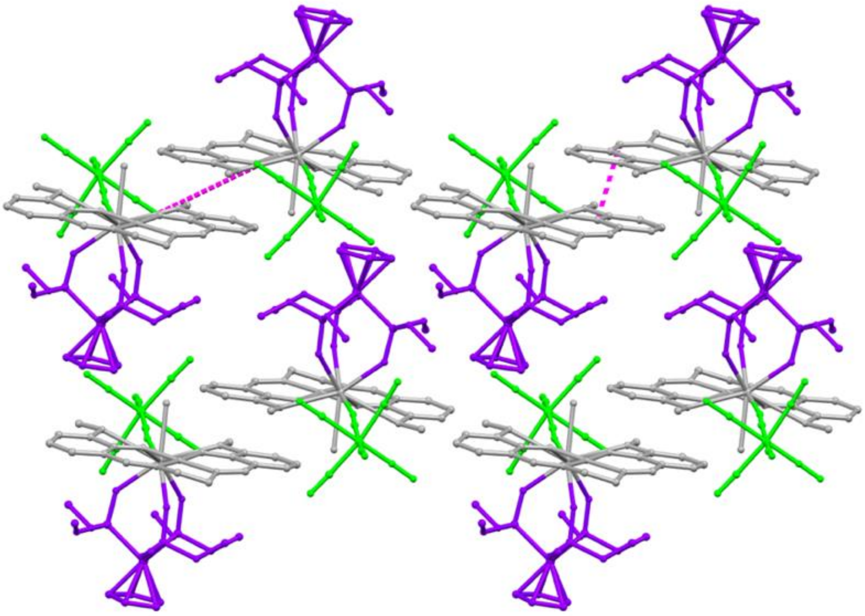

Figure S4. Crystal packing of ***1*** viewed along the b axis. The shortest intermetallic bond between two paramagnetic ions and the π-stacking between the phenyl rings of the valen units are highlighted in pink.

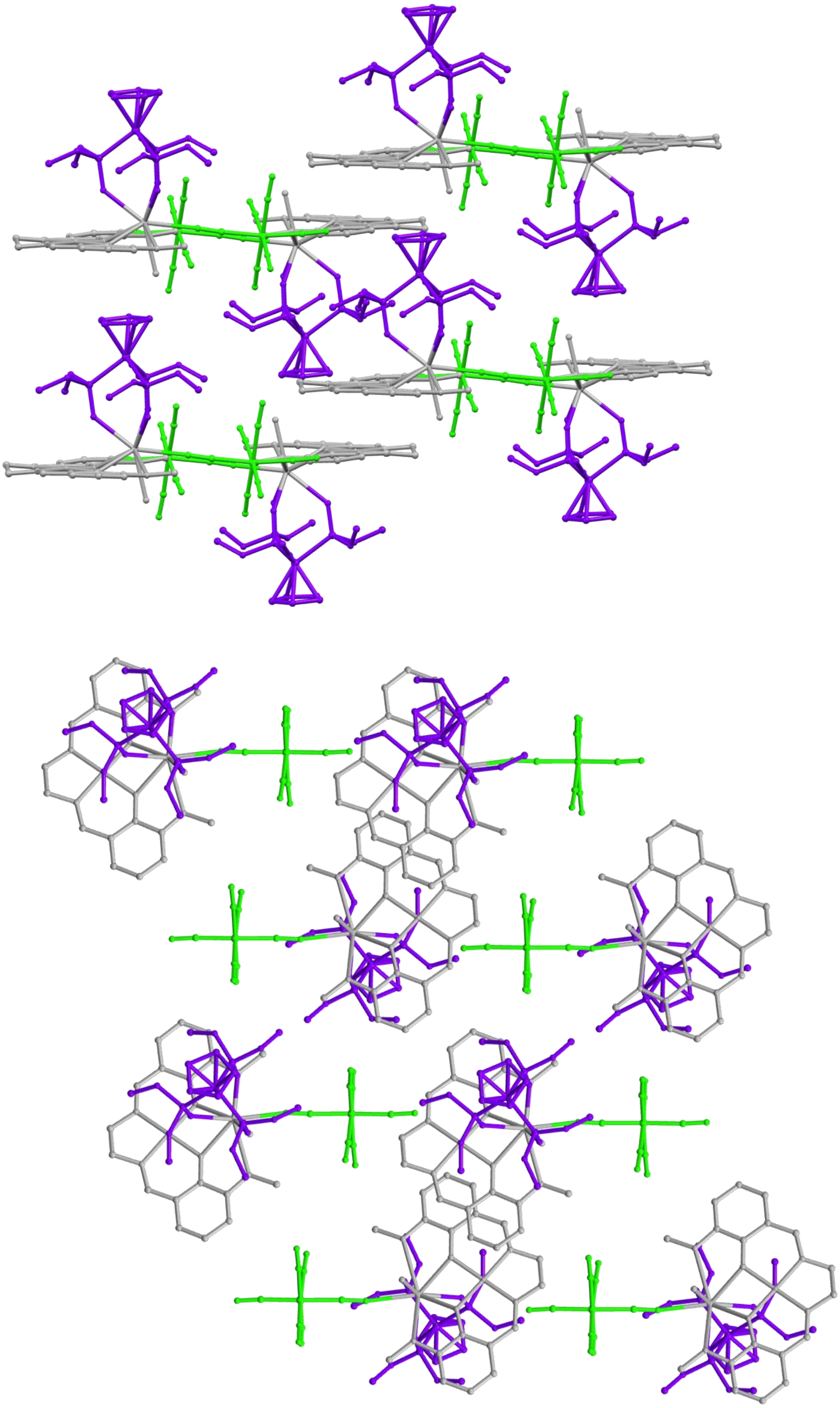

*Figure S5. Crystal packing of **1** viewed along the c (top) and b (bottom) axes.*

4. Magnetic Studies

4.1. Static magnetic investigations of Fe$_{NO}$CuLnCo complexes

4.1.1. Gadolinium complex Fe$_{NO}$CuGdCo (*1*)

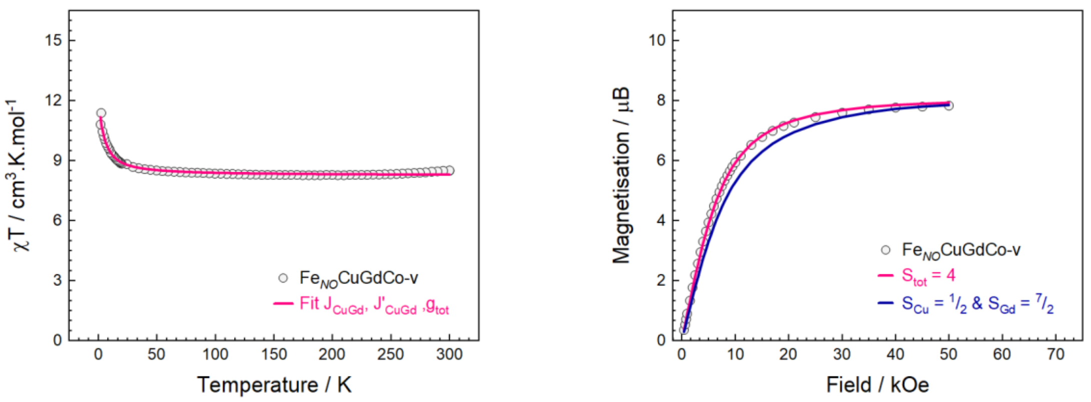

*Figure S6. Molar susceptibility and temperature product $\chi_m T$ thermal variation of **1** in the 2-300 K temperature range at 0.1k Oe (left) and field dependence of magnetisation of **1** in the 0-50k Oe field range at 2 K (right). Coloured lines represents the fits according to section 4.1.3.*

4.1.2. Fe$_{NO}$CuLnCo complexes

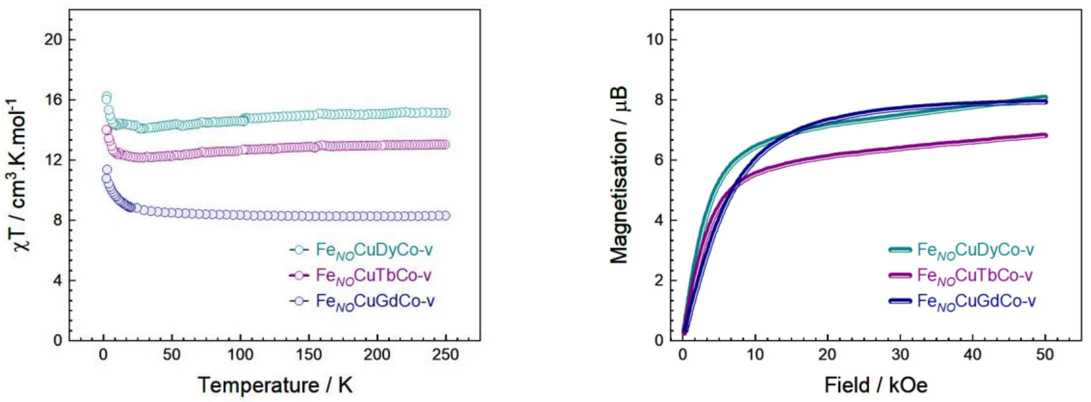

Figure S7. Molar susceptibly and temperature product $\chi_m T$ thermal variation of Fe$_{NO}$CuLnCo (Ln = Gd, Tb, Dy) in the 2-250 K temperature range at 0.1k Oe (left) and field dependence of magnetisation of Fe$_{NO}$CuLnCo (Ln = Gd, Tb, Dy) in the 0-50k Oe field range at 2 K.

4.1.3. Fits and simulations: equations

Fit thermal variation of the molar susceptibly and temperature product $\chi_m T$ using Hamiltonian $H2$ (spin-only intra- and inter-molecular interactions and g-factor)

$$\boldsymbol{H} = \boldsymbol{H}_{HDVV} + \boldsymbol{H}_{zeeman}$$

$$H_{HDVV} = -J_{CuGd} S_{Cu} \cdot S_{Gd}$$

$$H_{zeeman} = \beta g (S_{Cu} + S_{Gd}) H - zJ' S \cdot S$$

$$\boldsymbol{H} = -\boldsymbol{J}_{CuGd} \boldsymbol{S}_{Cu} \cdot \boldsymbol{S}_{Gd} + \boldsymbol{\beta g} (\boldsymbol{S}_{Cu} + \boldsymbol{S}_{Gd}) \boldsymbol{H} - \boldsymbol{zJ'S} \cdot \boldsymbol{S}$$

$$\chi = \frac{N\beta^2 g^2}{3k} \frac{\dfrac{\sum_S S(S+1)(2S+1)e^{\left(-E^{(0)}_{S_{Cu},S_{Gd}}/kT\right)}}{\sum_S (2S+1)e^{\left(-E^{(0)}_{S_{Cu},S_{Gd}}/kT\right)}}}{-\dfrac{zJ'\left(\sum_S S(S+1)(2S+1)e^{\left(-E^{(0)}_{S_{Cu},S_{Gd}}/kT\right)}\right)}{\sum_S (2S+1)e^{\left(-E^{(0)}_{S_{Cu},S_{Gd}}/kT\right)}}}$$

$$with\ S = S\_Cu + S\_Gd\ ;\ S\_Cu = 1/2\ \&\ S\_Gd = 7/2$$

$$and$$

$$e^{\left(-E^{(0)}_{S_{Cu},S_{Gd}}/kT\right)} = -\frac{J}{2}\left(S(S+1) - S_{Cu}(S_{Cu}+1) - S_{Gd}(S_{Gd}+1)\right)$$

$$\boldsymbol{\chi T = \frac{\dfrac{N\beta^2 g^2}{3k} \dfrac{28e^{-\frac{4J}{kT}} + 60}{7e^{-\frac{4J}{kT}} + 9}}{1 - zJ' \dfrac{N\beta^2 g^2}{3kT} \dfrac{28e^{-\frac{4J}{kT}} + 60}{7e^{-\frac{4J}{kT}} + 9}}}$$

Brillouin simulation of the field variation considering interacting Cu and Gd ions with a ground state spin value S_{tot}

$$y = 3{,}3249\mathrm{e}^{-5} g \left(\frac{2S_{tot}+1}{2}\right) e^{(2S+1)} x + \frac{e^{\left(-3{,}3249\mathrm{e}^{-5}(2S_{tot}+1)x\right)}}{e^{\left(3{,}3249\mathrm{e}^{-5}(2S_{tot}+1)x\right)}} - e^{\left(-3{,}3249\mathrm{e}^{-5}(2S_{tot}+1)x\right)} - \frac{g}{2} e^{\left(3{,}3249\mathrm{e}^{-5}x\right)} + \frac{e^{\left(-3{,}3249\mathrm{e}^{-5}x\right)}}{e^{\left(3{,}3249\mathrm{e}^{-5}x\right)}} - e^{\left(-3{,}3249\mathrm{e}^{-5}x\right)}\ with\ g = 2.00\ and\ S_{tot} = 4$$

Brillouin simulation of the field variation considering non interacting Gd and Cu ions

$$y = 3{,}3249\mathrm{e}^{-5} g \left(\frac{2S_{Cu}+1}{2}\right) e^{(2S_{Cu}+1)} x + \frac{e^{\left(-3{,}3249\mathrm{e}^{-5}(2S_{Cu}+1)x\right)}}{e^{\left(3{,}3249\mathrm{e}^{-5}(2S_{Gd}+1)x\right)}} - e^{\left(-3{,}3249\mathrm{e}^{-5}(2S_{Cu}+1)x\right)} - \frac{g}{2} e^{\left(3{,}3249\mathrm{e}^{-5}x\right)} + \frac{e^{\left(-3{,}3249\mathrm{e}^{-5}x\right)}}{e^{\left(3{,}3249\mathrm{e}^{-5}x\right)}} - e^{\left(-3{,}3249\mathrm{e}^{-5}x\right)} + 3{,}3249\mathrm{e}^{-5} g \left(\frac{2S_{Gd}+1}{2}\right) e^{(2S+1)} x + \frac{e^{\left(-3{,}3249\mathrm{e}^{-5}(2S_{Gd}+1)x\right)}}{e^{\left(3{,}3249\mathrm{e}^{-5}(2S_{Gd}+1)x\right)}} - e^{\left(-3{,}3249\mathrm{e}^{-5}(2S_{Gd}+1)x\right)} - \frac{g}{2} e^{\left(3{,}3249\mathrm{e}^{-5}x\right)} + \frac{e^{\left(-3{,}3249\mathrm{e}^{-5}x\right)}}{e^{\left(3{,}3249\mathrm{e}^{-5}x\right)}} - e^{\left(-3{,}3249\mathrm{e}^{-5}x\right)}$$

$$with\ g = 2.02\ and\ S_{Cu} = {}^{1}\!/_{2}\ and\ S_{Gd} = {}^{7}\!/_{2}$$

4.2. Dynamic magnetic investigations of Fe_{NO}CuLnCo (Ln = Tb, Dy) complexes

4.2.1. Terbium complex Fe_{NO}CuTbCo (*2*)

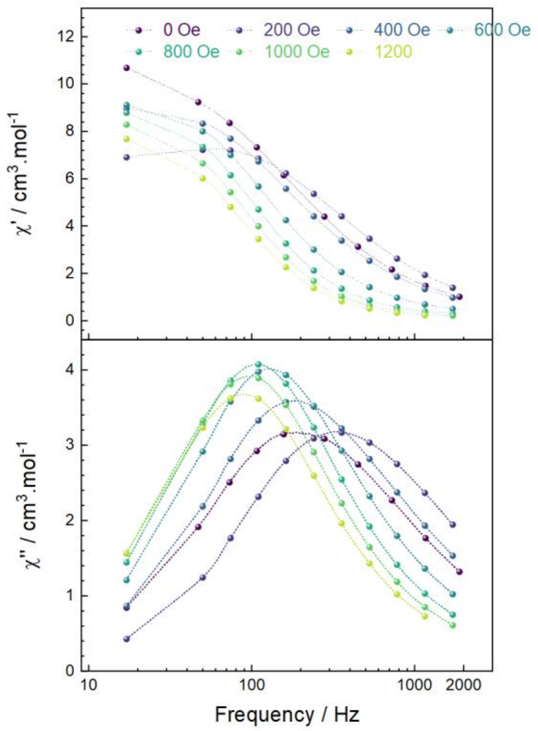

*Figure S8. χ′ and χ′′ frequency dependence of **2** in the 200-1.2k Oe applied field range at 2.1 K.*

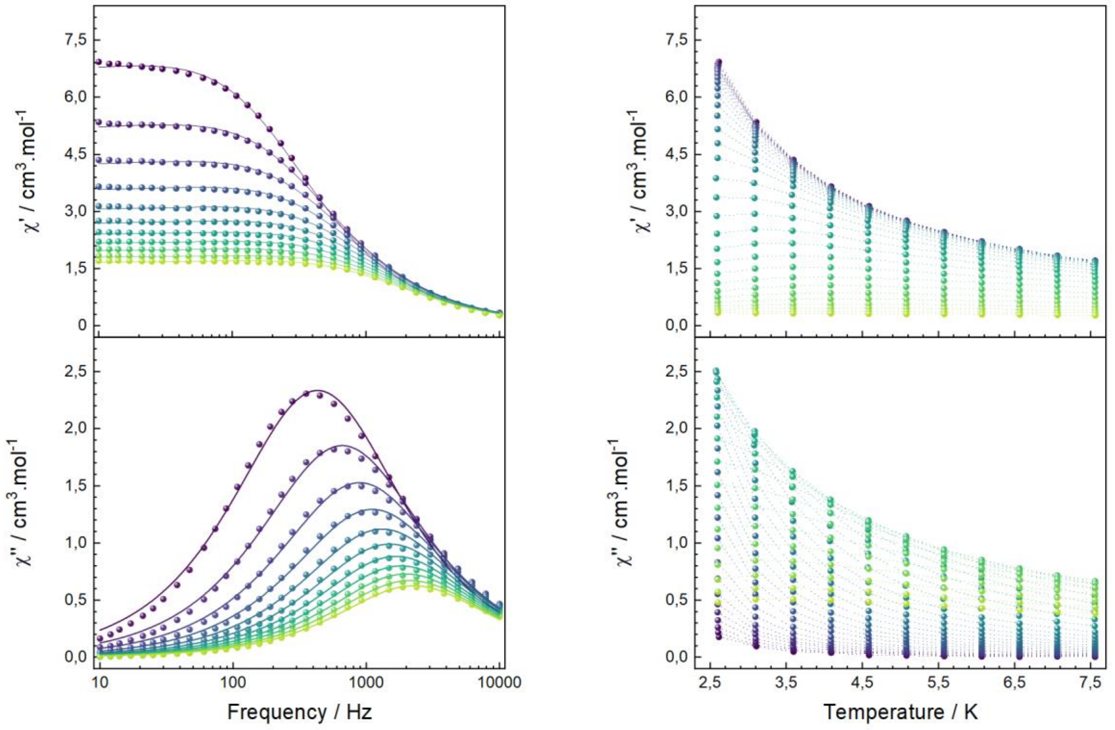

Figure S9. χ′ and χ′′ frequency (left) and temperature (right) dependence of 2 at zero-field in the 2.6-7.6 K temperature range. Solid lines represent fits to the data.

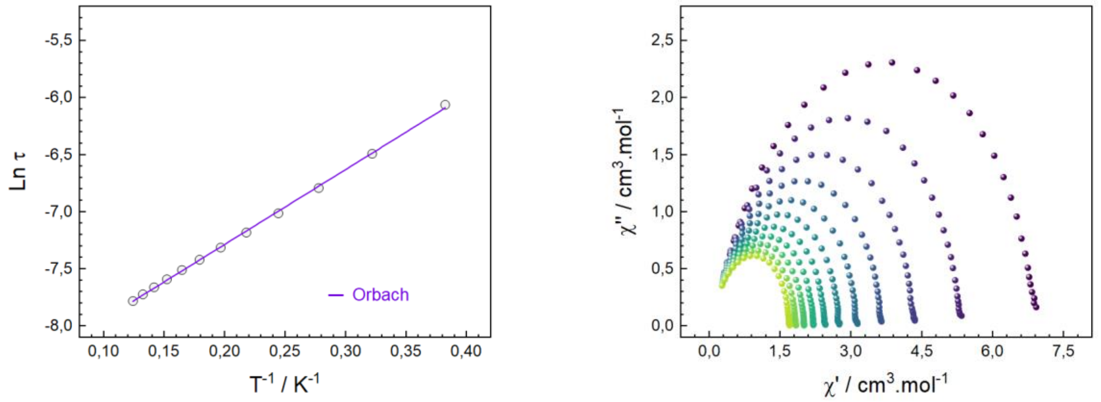

*Figure S10. Arrhenius plot of fitted relaxation times (left) and Cole-Cole plot in the 2.6-7.6 K temperature range (right) of **2** under zero applied dc field.*

4.2.2. Dysprosium complex Fe_{NO}CuDyCo (*2*)

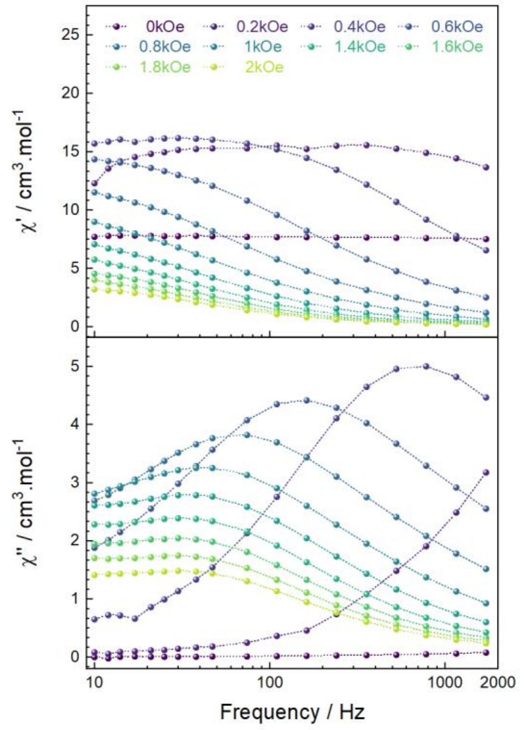

*Figure S11. χ' and χ'' frequency dependence of **3** in the 0-2k Oe field range at 2.1 K.*

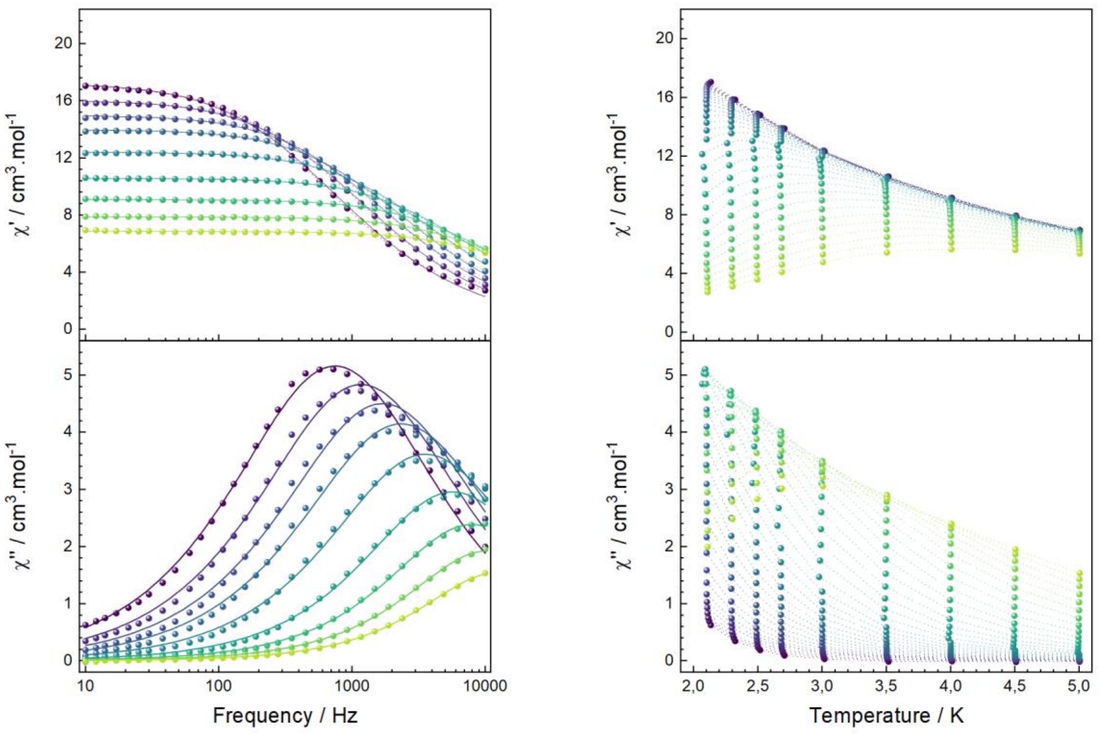

*Figure S12. χ′ and χ′′ frequency (left) and temperature (right) dependence of **3** under a 0.4k Oe applied field in the 2.1-5 K temperature range. Solid lines represent fits to the data.*

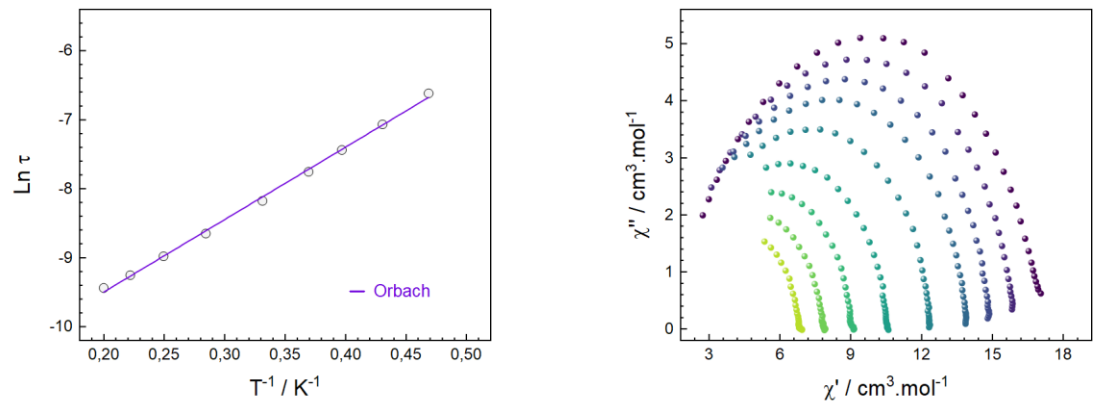

*Figure S13. Arrhenius plot of fitted relaxation times (left) and Cole-Cole plot in the 2.1-5 K temperature range (right) of **3** under 0.4k Oe applied dc field.*

5. Photo-switching studies

5.1. InfraRed Spectroscopy under light irradiation

The InfraRed studies under irradiation were collected over the range 4000-400 cm^{-1}. To ensure proper visibility, we have chosen to show spectra over the 2000-750 cm^{-1} range as the photo-induced spectral changes are observable in the NO band stretching region. Nevertheless, full range spectra are also provided to highlight both the absence of significant changes in the other regions over excitation and full reversibility after relaxation.

The experiment was conducted as followed. The sample was cooled down from room temperature to 10 K and spectra were collected in 50 K increments. Then, the sample was irradiated at 405 nm for at least 30 min – longer irradiation times did not lead to significant changes. In order to choose the most efficient excitation conditions, various excitation wavelengths were explored (405, 447, 476, 532 nm) and the evolution of the IR spectrum was followed in each case with different excitation laser power and different excitation duration. It is noteworthy that based on our experience with nitrosyl complexes photo-isomerism, long excitation duration and short excitation wavelength (near UV) frequently induce NO ligand release as an addition photo-induced undesired process. Photo-release may be avoided by using short exposures and visible wavelength in the blue range. Based on these preliminary tests, the optimum excitation conditions have been found with 405 nm excitation for 30 minutes duration. In all cases, the IR measurements on the photo-excited states have been performed after 30 minutes irradiation then shutting down the laser. The pellet sample on the cold finger of the cryostat is therefore completely thermalized during the IR measurement, and no laser heating effects can bias the experiment. Optical reversibility was investigated by irradiating the sample at 1064 nm for about 10 min following the same procedure. Finally, temperature reversibility was achieved by re-irradiating the sample at 405 nm for 30 min and by recording spectra while progressively increasing the temperature to RT.

5.1.1. Gadolinium complex Fe_{NO}CuGdCo (*1*)

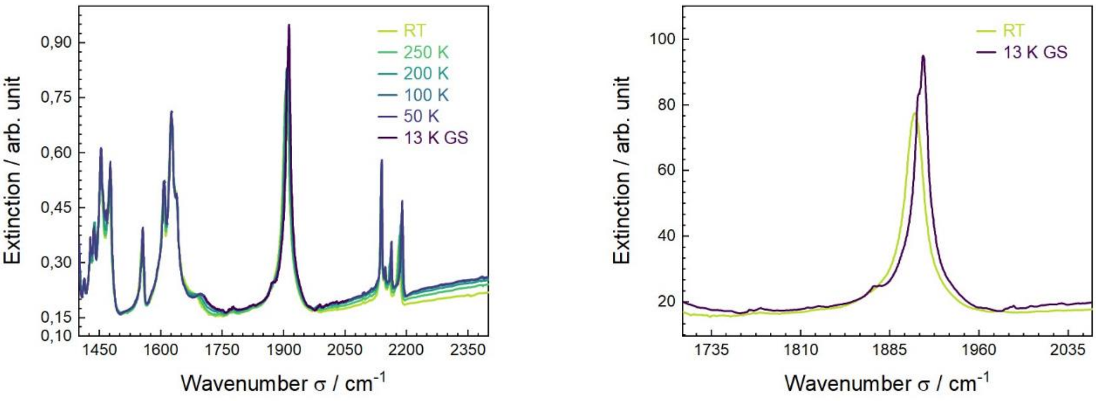

Figure S14. Spectra superimposition of Fe_{NO}CuGdCo (1) before excitation during cooling from 300 K to 13 K (left) and spectra superimposition of Fe_{NO}CuGdCo (1) before excitation at 13 K and 300 K (right).

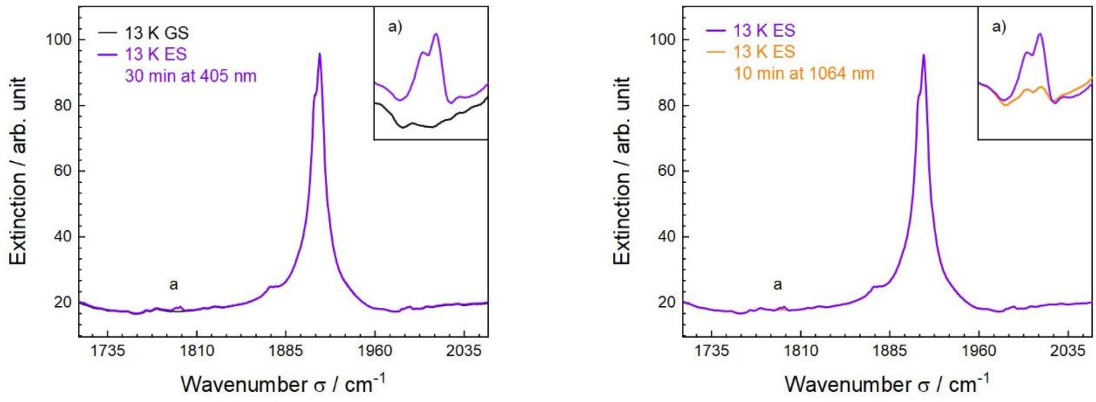

Figure S15. Spectra superimposition of Fe_{NO}CuGdCo (1) before excitation and after 30 min of irradiation at 405 nm (left) and spectra superimposition of Fe_{NO}CuGdCo (1) i) after 30 min of irradiation at 405 nm (ES) and after subsequent 10 min of irradiation at 1064 nm (right).

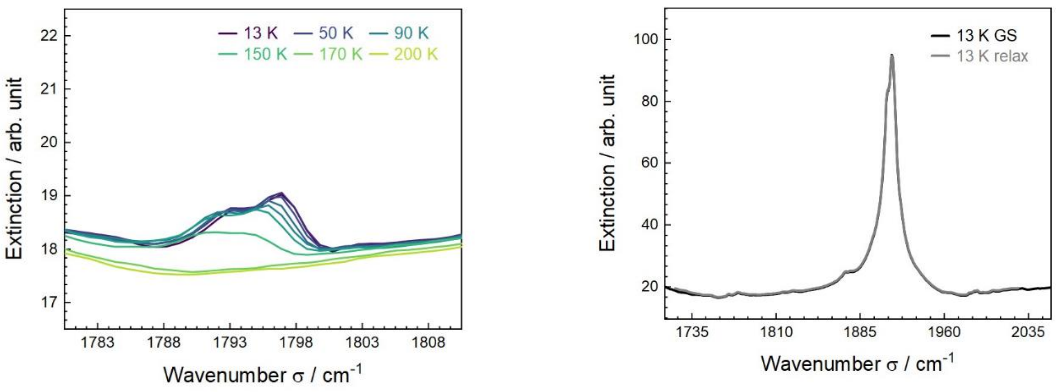

*Figure S16. Spectra superimposition of **1** after excitation (30 min, 405 nm) and further heating over the range of 13-300 K (left) and spectra superimposition of **1** before excitation and after raising the temperature to 300 K for thermal relaxation and further cooling to 13 K (right).*

5.1.2. Terbium complex $Fe_{NO}CuTbCo$ (*2*)

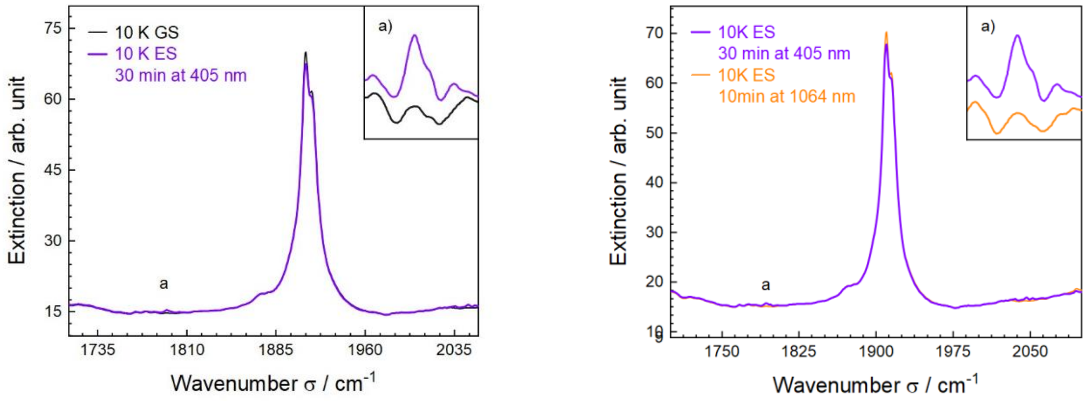

*Figure S17. Spectra superimposition of **2** before excitation and after 30 min of irradiation at 405 nm (left) and spectra superimposition of **2** i) after 30 min of irradiation at 405 nm (ES) and after subsequent 10 min of irradiation at 1064 nm (right).*

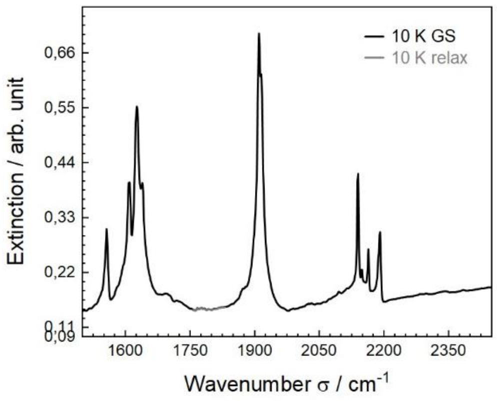

*Figure S18. Spectra superimposition of **2** before excitation and after raising the temperature to 300 K for thermal relaxation and further cooling to 10 K.*

5.1.3. Dysprosium complexes Fe_{NO}CuDyCo (*3*)

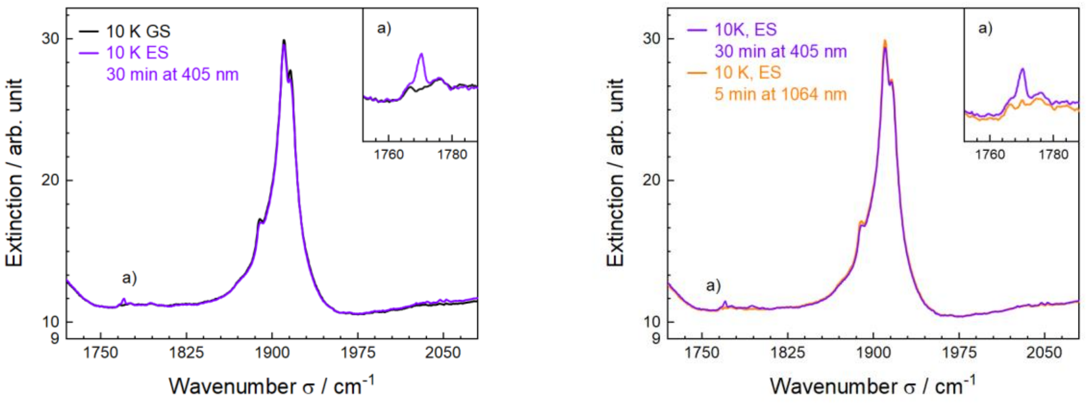

*Figure S19. Spectra superimposition of **3** before excitation and after 30 min of irradiation at 405 nm (left) and spectra superimposition of **3** i) after 30 min of irradiation at 405 nm (ES) and after subsequent 10 min of irradiation at 1064 nm (right).*

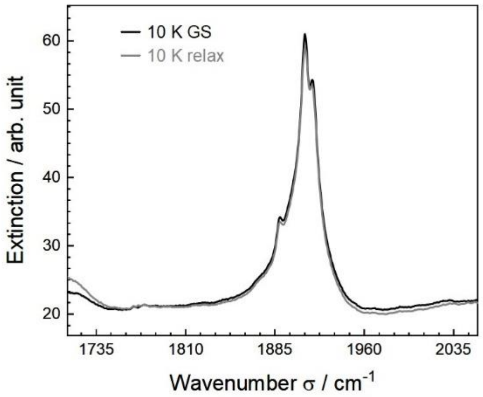

*Figure S20. InfraRed spectra superimposition of **3** before excitation at 10 K (GSs) and after 405 nm excitation at 10K and subsequent 5 min of irradiation at 1064 nm (optical reversibility).*

5.2. EPR measurements under irradiation

No significant changes on the spectra of Fe_{NO}CuLnCo (Ln = Gd, Tb, Dy) compounds were observed under irradiation at 4 K. It confirms the diamagnetic nature of the iron(II) metal ion in the photo-induced isonitrosyl isomer.

6. Literature

[1] Bruker. *APEX3* v2015.5-2; Bruker AXS Inc.: Madison, WI, USA, **2015**.

[2] Bruker. *SAINT* v8.34A; Bruker AXS Inc.: Madison, WI, USA, **2013**.

[3] Bruker. *SADABS* v2014/5; Bruker AXS Inc.: Madison, WI, USA, **2014**.

[4] O. V. Dolomanov, L. J. Bourhis, R. J. Gildea, J. a. K. Howard, H. Puschmann, *J Appl Cryst* **2009**, *42*, 339–341.

[5] G. M. Sheldrick, *Acta Cryst C* **2015**, *71*, 3–8.

[6] G. M. Sheldrick, *Acta Cryst A* **2015**, *71*, 3–8.

[7] T. Yi, Z. Wang, S. Gao, X. Chen, B. Ma, C. Liao, C. Yan, *Molecular Crystals and Liquid Crystals Science and Technology. Section A. Molecular Crystals and Liquid Crystals* **1999**, *335*, 211–220.

[8] M. Komine, K. Imoto, A. Namai, M. Yoshikiyo, S. Ohkoshi, *Inorg. Chem.* **2021**, *60*, 2097–2104.

[9] P. T. Manoharan, W. C. Hamilton, *Inorg. Chem.* **1963**, *2*, 1043–1047.

[10] A. Navaza, G. Chevrier, P. M. Alzari, P. J. Aymonino, *Acta Cryst C* **1989**, *45*, 839–841.

[11] D. F. Mullica, E. L. Sappenfield, D. B. Tippin, D. H. Leschnitzer, *Inorganica Chimica Acta* **1989**, *164*, 99–103.

[12] P. T. Manoharan, H. B. Gray, *J. Am. Chem. Soc.* **1965**, *87*, 3340–3348.
